# Experience of uncertainty in prostate cancer: A qualitative study

**DOI:** 10.1371/journal.pone.0334180

**Published:** 2025-10-13

**Authors:** Yingna Li, David Gillanders, Anne Finucane

**Affiliations:** 1 Health in Social Science, The University of Edinburgh, Edinburgh, United Kingdom; 2 Marie Curie Hospice Edinburgh, Edinburgh, United Kingdom; University of Rome 'Foro Italico': Universita degli Studi di Roma Foro Italico, ITALY

## Abstract

**Background:**

Prostate cancer is characterised by significant uncertainty, yet men’s lived experience of uncertainty in this context remain underexplored. Existing research has primarily focused on uncertainty related to informational needs, the pre-treatment phase, or men under active observation. Little attention has been paid to uncertainty that extends beyond informational gaps or affects those who have undergone active treatment.

**Objective:**

This study aimed to address this gap by exploring how men experience uncertainty throughout the prostate cancer journey. Gaining such insight is essential for informing more holistic and responsive care.

**Methods:**

Individual semi-structured interviews were conducted with 12 men diagnosed with prostate cancer who were either undergoing or had completed active treatment. Data were analysed using reflexive thematic analysis.

**Results:**

Four overarching themes were developed: (1) Aspects of uncertainty, including ambiguity surrounding the cancer trajectory, interpersonal relationships, and the future; (2) Initial responses to uncertainty, including emotional distress and avoidant behaviours; (3) Managing uncertainty, including strategies used to cope and adapt; and (4) Posttraumatic growth, highlighting positive changes attributed to navigating prostate cancer and its uncertainty.

**Conclusions:**

Uncertainty is a pervasive and enduring aspect of the prostate cancer experience, extending beyond diagnosis into treatment and survivorship. It complicates psychological adjustment and calls for adaptive management. Support interventions should address not only informational but also emotional and relational dimensions of uncertainty, promoting openness and acceptance, perspective-taking, values-driven living, and dyadic adjustment.

## Introduction

Uncertainty is a universal experience characterised by a feeling of incomplete understanding due to insufficient, ambiguous, unreliable, or even conflicting information [1–3]. Mishel’s [2] theory of illness uncertainty identified four features of the illness experience that give rise to uncertainty: complexity (e.g., multiple symptoms or treatments), unpredictability (e.g., uncertain treatment outcomes, disease progression or recurrence), ambiguity (e.g., unclear or conflicting information), and lack of information.

Illness uncertainty, if poorly addressed, can lead to psychological distress [2,4], particularly in conditions such as cancer [5,6]. Uncertainty may be especially salient in individuals diagnosed with metastatic cancer of unknown primary origin, who must contend with uncertainty regarding both diagnosis and prognosis [7]. Even for those with a clearer medical diagnosis, predicting the prognosis of cancer can be challenging [8], often leaving patients overwhelmed by uncertainty throughout their illness [6,9]. Uncertainty can complicate the treatment decision-making process [10], lead patients to defer decisions to their clinicians [11], or result in decisional conflict [12]. Uncertainty may persist beyond diagnosis and treatment, extending across the illness trajectory and continuing to fuel psychological distress. Fear of cancer recurrence, for example, can persist long after treatment and is heightened by uncertainty about treatment effectiveness and future health outcomes [13,14]. A recent meta-analysis also yielded a moderate-sized negative correlation between illness uncertainty and social support in adults with cancer [15]. As such, uncertainty can profoundly impact an individual’s adaptation to living with a serious chronic condition.

Uncertainty plays a particularly salient role in prostate cancer, the most commonly diagnosed cancer in males in the United Kingdom (UK), with approximately 55,100 new cases each year [16]. Prostate cancer presents men with a wide range of trajectories, from slow-growing tumours manageable with less damaging treatments to aggressive forms requiring urgent interventions [17]. This variability creates uncertainty concerning treatment effectiveness, side effects, recurrence, and impacts on relationships and support [18,19]. Experiences of uncertainty are often associated with psychological difficulties. Quantitative research has found associations between uncertainty with heightened anxiety and worry, and poorer quality of life [19–21]. Moreover, uncertainty can disrupt men’s self-perception, complicate their understanding of the cancer journey, interfere with decision-making, and lead to greater avoidant coping [21–23]. In a large sample of 978 men with prostate cancer, over 20% reported a moderate to high unmet need for support in addressing uncertainty about the future [24].

While previous research has highlighted the relevance of uncertainty in prostate cancer, men’s lived experience is not well articulated. Most existing research has centred on informational needs and common concerns, such as diagnostic testing, treatment options, prognosis, and side effects management [25–28]. Although not always explicitly framed in terms of uncertainty, these findings can be understood as addressing aspects of it, through the lens of reducing informational gaps. However, treating uncertainty as a problem of insufficient information risks overlooking its emotional, relational, and existential dimensions. Studies that examine uncertainty beyond informational need have largely centred around men in the pre-treatment phase or under active observation (e.g., active surveillance or watchful waiting) [19,23,29–33], with little attention to those who have undergone active treatment. A recent qualitative study began to address this gap by exploring post-treatment experiences of uncertainty [34], but it focused narrowly on clinical uncertainty in terms of prostate-specific antigen monitoring and fears of recurrence. Less is known about how men navigate uncertainty emotionally and psychologically across the broader prostate cancer trajectory.

The present study aimed to investigate how men perceive and respond to uncertainty related to prostate cancer throughout the cancer trajectory. An in-depth and comprehensive understanding of uncertainty in men’s experience is essential for understanding how uncertainty is communicated and managed in clinical settings. Such evidence may inform the development of supportive interventions for men with prostate cancer and their families, as well as guide future research on psychological care in this population.

## Methods

### Design

A qualitative design with individual semi-structured interviews analysed using reflexive thematic analysis [35]. Given the exploratory nature of the study, an inductive interpretative approach with a phenomenological lens was adopted, capturing both semantic and latent meanings in participants’ accounts. Imposing no predetermined theoretical or conceptual framework, this approach allows the data to guide the analytical structure [36].

### Patient and public involvement

Three patient representatives with lived experience of prostate cancer were identified through Prostate Scotland and a peer support network through LinkedIn. They confirmed the relevance of the research purpose and advised on research design, participant recruitment, early report drafts and dissemination.

### Participants

Participants were eligible if they had received a diagnosis of prostate cancer, were 18 years or older, and resided in the UK. A poster and a link to participant information sheet were distributed via social media (e.g., Facebook and Twitter/X) and relevant online forums (e.g., Prostate Cancer UK, Prostate Scotland, and Prostate Cancer Research). To recruit a more diverse sample, we advertised our study in online groups relevant to sexual and ethnic minority men (e.g., Reddit). Participants were recruited between 1^st^ May and 26^th^ November 2024. Upon providing online informed consent, participants completed a brief demographic questionnaire. Participants were given the option to meet in person at the university campus, via a Teams meeting or over the phone. There was no prior relationship between participants and researchers.

Prior methodological reviews noted that it could be problematic to determine sample size before data collection and analysis in qualitative research and researchers are suggested to appraise the data richness or adequacy in relation to the intrinsic properties of a specific study [37,38]. Given this, and in line with the feature of reflexive thematic analysis, we adopted the conceptual model of information power to guide our sample size considerations. According to this model, the more information a sample holds, the fewer participants are required [39]. Recruitment and analysis occurred in parallel, whereby we iteratively appraised the information power of the sample before deciding the final sample size.

Specifically, the data richness was evaluated against the parameters proposed by the information power model [39]: (1) *Research aim*: for the current study, it was neither too broad nor too narrow; (2) *Sample specificity*: the eligible participants are considered with characteristics (i.e., experience of prostate cancer and uncertainty) that are sufficiently specific for the research aim; (3) *Application of established theory*: no prior theories guided the design or data analysis; (4) *Analytical strategy*: we aimed to obtain a balance between within-case and cross-case analysis, with the intention to capture the uniqueness of individual experiences and the commonalities across men with prostate cancer, and (5) *Quality of researcher-participant dialogues*: the interview conversations were generally rich, in-depth, and reflective, offering detailed descriptions of participants’ lived experiences.

We assessed emerging patterns and judged that by the twelfth interview, no substantively new themes were arising, suggesting thematic sufficiency. While we did not aim for saturation in the traditional sense, we judged that the sample held sufficient information power to address our research aim meaningfully.

### Data collection

Semi-structured individual interviews were conducted in person or online (via Teams) by the first author. An interview protocol, developed by the research team with input from patient representatives was used (S1 File). Participants were asked open-ended questions about their general experience of prostate cancer, their experience of uncertainty, and how they managed it. Field notes were taken during the interviews to facilitate later data analysis. The interviews were audio recorded.

### Data analysis

The interviews were transcribed verbatim and anonymised within two weeks of completion by the first author. Identifying information (e.g., names, places, employers) were removed or anonymised during transcription. De-identified data were stored securely on encrypted university servers, and only members of the research team had access.

Interview transcripts were analysed using NVivo (Version 14), a widely used software for qualitative research that facilitates efficient organisation and management of qualitative data. It enables systematic line-by-line coding, supports rapid retrieval of coded extracts, and provides a clear overview of developing codes and their definitions.

Reflexive thematic analysis was undertaken following Braun and Clarke’s [35] six-stage guide: (1) familiarisation with data through repeated readings of transcripts and reflective notetaking, (2) generation of initial codes through systematic, line-by-line coding to capture both sematic content (i.e., what was explicitly said) and early interpretations of latent meaning (e.g., how or why something was said), (3) generation of initial themes by clustering codes based on shared meaning and relevance to the research aim, (4) iterative review and refinement of themes to ensure they were logically coherent, reasonably distinct from one another, and well supported by rich data extracts, (5) labelling and defining the final themes and subthemes, and (6) producing the final report. The analysis was primarily data-driven and inductive, with codes and themes developed from the content of participants’ accounts rather than from pre-determined theoretical frameworks.

To enhance the rigour and depth of analytical process, the first and third authors independently coded an initial subset of transcripts and met regularly to discuss and refine the coding approach. Differences identified were not treated as disagreements to be resolved but as opportunities to consider alternative interpretation of the data, consistent with the principle of reflexive thematic analysis. The remaining transcripts were coded by the first author, who engaged in iterative analysis by regularly revisiting earlier transcripts in light of emerging patterns and developing novel insights.

Themes were actively constructed through the researcher’s subjective interpretation of the data rather than passively ‘discovered’. We paid attention to both participants’ explicit accounts (semantic level) and the underlying meanings or values inferred from their narratives (latent level). In some cases, the interpretations of latent meanings drew on field notes taken during interviews to capture non-verbal cues such as facial expression or physical gestures that offered additional contextual information.

A summary table of themes, subthemes, codes, and illustrative quotes is provided as a supporting material (S2 File) which presents a clear overview of the analytic process, including how initial codes were clustered into subthemes and then organised into overarching themes.

### Reflexivity

Reflexivity was an integral part of the study. The research team met regularly throughout the study to reflect on the process, beginning with discussions of interviewing techniques aimed at improving participant engagement and data depth. As analysis progressed, the team continued to assess the data quality in terms of both depth and breadth, using these insights to inform subsequent interviews and analyses.

The first author conducted all interviews and led the analysis. She is a middle-aged Chinese doctoral researcher with training in qualitative methods and a research focus on men’s psychological adjustment to prostate cancer. To support reflexive practice, she kept a reflexive journal throughout data collection and analysis. This journal served as a tool for critically appraising how her own background and values might have influenced the research process. It documented her evolving assumptions, emotional responses, and interpretive decisions, as well as reflections.

Reflexivity was actively integrated into data interpretation through repeated use of the journal to interrogate emerging insights. For example, early in the analysis, the first author initially interpreted participants’ references to their partners as evidence of strong emotional support. However, upon deeper reflection and re-reading, she noted the absence of emotional dialogue in these descriptions and began to consider that some expressions of support were more instrumental than emotional in nature. This realisation prompted her to explore this distinction more explicitly in subsequent interviews, ultimately contributing to the development of the theme on relational uncertainty.

The first author also reflected on how her cultural background and assumptions shaped her interpretation. For instance, she initially perceived some participants as unusually emotionally open. However, recognising that her expectations were informed by Chinese cultural norms, where emotional restraint is often strongly valued, she revisited the data with greater sensitivity to indirect or culturally specific forms of emotional disclosure. This helped refine her interpretations and avoid misattributing emotional openness or distance.

While the analysis was primarily inductive and grounded in participants’ accounts, we acknowledge that the researchers’ prior knowledge and theoretical sensitivities, including familiarity with concepts such as uncertainty and coping, inevitably influenced the interpretive process. This reflexive stance is consistent with the principles of reflexive thematic analysis, which recognises that researchers bring their own perspectives and experiences into the analytic process [35].

In addition to reflecting on positionality and interpretation, we also considered how aspects of study design might influence data quality. For example, we reflected on the potential influence of different interview modalities (i.e., in-person or online) on interviewer-participant rapport and participant disclosure. We initially expected that in-person interviews may yield greater rapport and more emotionally detailed disclosure. However, participants in both formats appeared open and reflective in discussing their experiences, including emotionally difficult topics. The quality and emotional depth of the data obtained online were comparable to those from the in-person interviews. While we cannot entirely rule out subtle differences, we believe that the flexibility of format helped accommodate participants’ preferences and comfort and did not compromise the quality or emotional depth of the narratives.

### Reporting

We used the consolidated criteria for reporting qualitative research (COREQ) [40] to ensure a comprehensive report.

### Ethical approval

Institutional ethical approval was obtained from the School of Health in Social Science Research Ethics Committee of the University of Edinburgh (Reference: 23–24CLPS005) on 16 November 2023. All participants completed an online informed consent form to indicate their willingness to participate in the current study.

## Results

### Sample characteristics

Twelve participants with a mean age of 66 years (range 52-80) were recruited. Two interviews were conducted in person on campus, and ten via Teams. Interviews typically lasted between 60 and 75 minutes. All participants identified as White British and heterosexual. Most were married or in an intimate relationship (92%), held higher education degrees (75%) and lived in Scotland (58%). Time since diagnosis ranged from three months to thirteen years and two months, with a median of twenty-three months. Two participants had advanced prostate cancer at diagnosis, while the remaining ten had localised cancer. All had received one or more forms of active treatments, with one initially on active surveillance. At recruitment, eight participants had completed their primary treatment, while the remaining four were undergoing active treatment. Participant demographics at the group and individual levels are presented in Tables 1 and 2, respectively.

**Table 1:** Sociodemographic and clinical characteristics of participants characteristics at the group level (N = 12).

Characteristic	n	%
**Age**: Mean 66 (SD 6.34; range 52–80)		
**Ethnicity**		
White British	12	100
**Place of residence**		
England	5	41.7
Scotland	7	58.3
**Sexual orientation**		
Heterosexual or straight	12	100
**Highest education**		
High school diploma or equivalent	2	16.7
Bachelor’s degree	4	33.3
Master’s degree	5	41.7
Prefer not to say	1	8.3
**Employment status**		
Retired	6	50
Employed full-time	3	25
Employed part-time	2	16.7
Unemployed	1	8.3
**Relationship status**		
Partnered (Mean of relationship length in years 27.18, SD 16.59; range 5–40)	11	91.7
Unpartnered	1	8.3
**Months since diagnosis**: Mean 41.50 (SD 44.46; range 3–158)		
**Cancer stage at diagnosis**		
Localised or early	10	83.3
Metastatic or advanced	2	16.7
**Primary treatment complete**		
Yes	8	66.7
No	4	33.3
**Treatment modality**		
Hormone therapy alone	1	8.3
Prostatectomy or surgery alone	4	33.3
Radiotherapy and hormone therapy	4	33.3
Hormone therapy, radiotherapy and surgery	2	16.7
Active surveillance, hormone therapy, radiotherapy and surgery	1	8.3

N.B. SD = Standard deviation.

**Table 2:** Sociodemographic and clinical characteristics of participants at the individual level (*N* = 12).

Assigned ID	Age	Place of residence	Education background	Employment status	Relationship status	Relationship length (in years)	Months since diagnosis	Cancer stage at diagnosis	Treatment received	Primary treatment complete
P1	80	Scotland	Master’s degree	Retired	Partnered	17	32	Advanced	Hormone therapy	No
P2	71	England	Master’s degree	Retired	Partnered	5	19	Localised	Surgery	Yes
P3	69	Scotland	Bachelor’s degree	Retired	Partnered	51	32	Localised	Hormone therapy; Radiotherapy	Yes
P4	65	Scotland	Bachelor’s degree	Retired	Partnered	8	72	Localised	Hormone therapy; Radiotherapy; Surgery	Yes
P5	64	Scotland	Master’s degree	Employed part-time	Partnered	7	17	Localised	Surgery	Yes
P6	65	England	Bachelor’s degree	Retired	Partnered	48	3	Localised	Hormone therapy; Radiotherapy	No
P7	71	Scotland	High school diploma or equivalent	Retired	Partnered	36	96	Localised	Surgery	Yes
P8	65	England	Prefer not to say	Employed full-time	Partnered	33	21	Localised	Hormone therapy; Radiotherapy	Yes
P9	65	England	Bachelor’s degree	Employed full-time	Partnered	38	158	Localised	Active Surveillance; Hormone therapy; Radiotherapy; Surgery	Yes
P10	64	Scotland	Master’s degree	Employed full-time	Partnered	16	25	Localised	Surgery	Yes
P11	65	England	High school diploma or equivalent	Employed part-time	Partnered	40	11	Localised	Hormone therapy; Radiotherapy	No
P12	52	Scotland	Master’s degree	Unemployed	Single	/	12	Advanced	Hormone therapy; Radiotherapy; Surgery	No

N.B. Assigned ID were created for anonymity. All participants self-identified as White British and heterosexual

### Overview of themes

**Fig 1:**
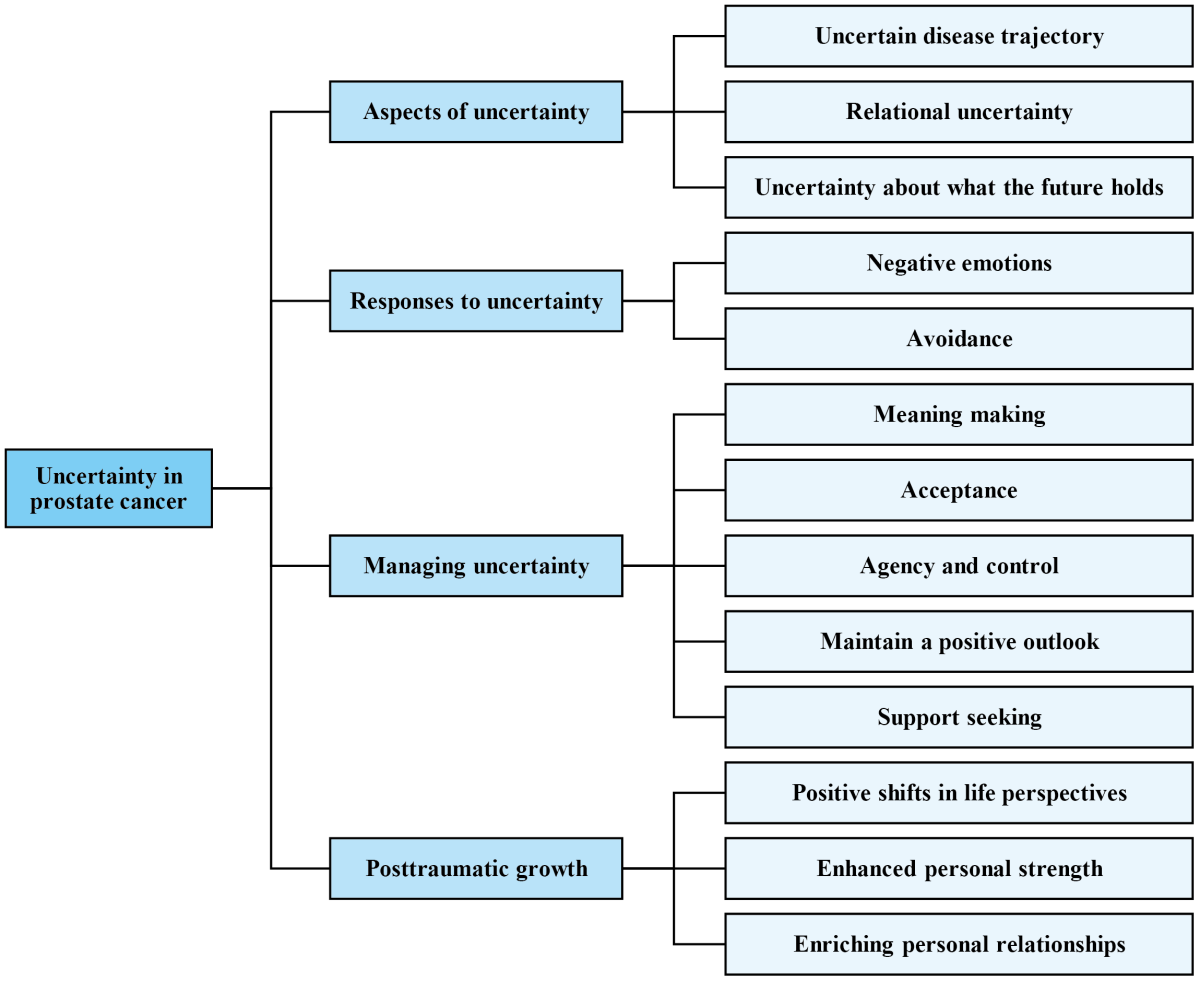
Theme structure.

Four overarching themes and 13 sub-themes were generated. The first overarching theme (aspects of uncertainty) reflected the various uncertainties perceived by participants, the second (reactions to uncertainty) showed initial negative emotional and behavioural reactions, the third (managing uncertainty) captured participants’ strategies, and the last (posttraumatic growth) concerned positive changes participants perceived through their experiences of cancer and uncertainty. The theme structure is presented in Fig 1. Further descriptions and illustrative quotes for each sub-theme are presented in text below.

### Overarching Theme 1: Aspects of uncertainty

#### Uncertain disease trajectory.

All participants described uncertainty related to the cancer trajectory, including diagnosis, symptoms, treatment options, outcomes, side effects, functional recovery, and the risk of recurrence. They particularly grappled with predicting treatment effectiveness and prognosis, as well as making treatment decisions. Uncertainty intensified when genuine shared decision-making was absent, leaving participants feeling unsupported or solely responsible for complex treatment choices.

“I think the part that really challenges … is you’ll get all the information you want to make a decision, but you have to make that decision. No one’s gonna make that decision for you.” (P10)

While most participants prioritised survival, some expressed a strong desire to avoid adverse effects when considering treatment options.

“I began to wonder would it be better if I just didn’t have the radiotherapy but had a good quality of life for two years and just accept I would die after that. I wondered if that might be a better life than living with added long term side effects from radiotherapy.” (P12)

A minority of participants recognised that individual treatment outcomes may not be accurately predicted using population-level clinical statistics. While most feared falling on the less favourable end of the spectrum, one participant with terminal cancer viewed uncertainty as a source of hope.

“The issue is about real-life, real-world data.... We know the statistical probabilities from the tests, but we also know that the participants in the tests are very different from the real life, the patient profile…. It was a relief to understand the limitations of the clinical trial outcomes as predictors of my life expectancy as an individual.” (P1)

#### Relational uncertainty.

Nearly all participants reported experiencing uncertainty in their interpersonal relationships. Those with partners were unsure whether their partner truly understood the implications of prostate cancer or could provide support they needed. They were therefore concerned about the stability of their relationship, especially considering the impact of erectile dysfunction on their role as an intimate partner.

“I’ve been chemically castrated. I was told that from day one.… it’s an issue for the partner to understand. And my partner does and doesn’t but does in part. Yeah. So how do we resolve and find new ways, you know, of relating physically. And that’s still an ongoing struggle.” (P1)

Some participants feared causing distress to their loved ones and therefore withheld concerns, avoiding difficult, though potentially meaningful, conversations.

“I should have said, but you know, how do you feel about this? Shouldn’t I?” (P11)

Withholding open conversations about cancer-related issues often impeded mutual understanding, heightened uncertainty about relationship stability, and made coping together harder.

“Never had any kind of deep conversations… I’m thinking I never…. really gave a thought that it’s not just me that’s going through this.” (P10)

For one participant not currently in a relationship, the diagnosis introduced uncertainty about future opportunities for intimacy and family life.

“I was single when I caught the diagnosis. So you think to yourself, well, you’re single forever, like you know who’s gonna want to have a relationship with me now.” (P12)

Some employed participants worried that their colleagues might perceive or treat them differently, even when their responses were intended to be caring or supportive.

“You don’t know how other people are going to react. You don’t want to appear weak or dependent or attention seeking.… Treat me as I wanted to be treated, as if none of this had ever happened. Give me the choice. If I can’t do what you want me to do, I will tell you. What I don’t want is anybody making the decision for me without telling me.” (P9)

#### Uncertainty about what the future holds.

Most participants described a future-oriented, existential uncertainty, questioning how long they would live and what kind of life they could expect in the years ahead.

“I’ve had good treatment. I wonder how long I will last.” (P10)

This uncertainty was characterised by a tension between hopes for functional recovery and the disappointing reality of persistent functional impairments. For many, this struggle intensified when their lived experience failed to match expectations set by medical advice.

“I think for me the biggest psychological problem was I had an expectation from what I’ve been told that. ‘You will have a bit of incontinence after the operation, but within three to six months, that’ll be fine, and you won’t have any problems.’ And that was never the case.… That I found really hard.” (P2)

One participant with advanced prostate cancer described how unexpected cancer recurrence introduced further uncertainty and led to a loss of trust in healthcare professionals.

“But then suddenly, nine months later, my PSA [prostate-specific antigen] score suddenly shot up. And at that point, I lost, I’d be honest, I lost some faith in the urologist…. the surgeon’s view was that there was a 94% chance that I would be cancer free for 10 years. Unfortunately, within a year the PSA score has started rising again.” (P9)

In addition to health-related concerns, uncertainty about the future affected participants’ ability to make long-term plans around finances, careers, and family activities. Some described a sense of powerlessness and relational strains as they struggled to balance hope with the unknown.

“If I’ve got 10 years to live, we can do this, this, this and this and this…. But if I’ve got 20 years left to live, plus I might have some care home at the end of my life, what does that mean financially? And it’s the great unknown, isn’t it? I know you asked about a health question, but to me the financial and the health thing are so inextricably linked.” (P11)

### Overarching Theme 2: Reactions to uncertainty

#### Negative emotions.

Shock and disbelief were common reactions to the diagnosis, with many participants initially struggling to make sense of what it meant or what to do next.

“… it was a tremendous shock. Really awful, really, really awful. Just very difficult to accept what was happening…. enormous events vthat are very hard to process, very difficult to deal with.” (P12)

Some participants described profound sadness and emotional overwhelm, feelings they rarely or never had before, and noted that it was so overwhelming that they could not discern whether it stemmed from fear.

“And I was just, I remember, just crying in the car, really. Almost like a big release. You know, I can’t even think why. If I was scared? … And I remember the day that was absolute torrential rain. I thought, well, I gotta stop crying or I won’t be able to see where I’m going.” (P10)

While the intense feelings of shock and sadness typically diminished over time, anxiety associated with uncertainty often persisted and could be reactivated in certain contexts.

“There’d still be the anxiety of cancer in the background.” (P3).

Some also experienced anger, which was often linked to concerns about how their illness may affect their loved ones.

“When I got the diagnosis with prostate cancer, all I felt I was angry with myself because I put everybody I love in a position where they had to worry.” (P8)

The previously noted tension between expectations and reality further contributed to anger and frustration.

“…that become anger after a while, when you realise, no, I haven’t been told the truth here.” (P5)

#### Avoidance.

Perceived uncertainty led some participants to withdraw or delay engagement with their condition. Even before diagnosis, ambiguous symptoms often triggered feelings of uncertainty and subsequent avoidant behaviours. One participant, for example, ignored haematuria for three years and only sought medical attention after being prompted by his partner, resulting in a late diagnosis.

“I knew there was something wrong three years before diagnosis, but I just said, well, it’ll go away. It’ll just go away.” (P8)

During the treatment decision-making phase, unclear treatment risks and outcomes contributed to indecision and delays in initiating treatment.

“…that process of deciding what to do took about four months… that was unnecessarily long. And nobody really benefited from that because that delayed my treatment….” (P12)

Some described becoming preoccupied with their thoughts, caught in cycles of rumination they struggled to break.

“…I was preoccupied with thinking about it every day. I was thinking about it all the time. It was constant thinking and ruminating on something that I didn’t want to have.” (P3)

### Overarching Theme 3: Managing uncertainty

#### Meaning making.

All participants described making sense of their experiences to restore meaning. Some reflected on how their pre-existing values, beliefs, coping styles, and past experiences shaped their responses to prostate cancer and its associated uncertainty.

“I have gone through my life thinking that everything and anything that goes wrong for me, for my family or around me is my fault…. And I think that’s informed my attitude to cancer.” (P9)

Some participants engaged in positive framing, viewing moments of uncertainty, introspection, and even pessimism not as distressing but as integral to their personal experiences. This process enabled reflection and growth without letting negative experience define their overall outlook on life.

“It’s not necessarily been at all negativities by any means. There is a positive aspect to this process that I’ve been through.…. I allow myself to have these moments of uncertainty and moments of introspection and moments of pessimism, if you like. But that can’t determine what your life is going to be like….” (P4)

All participants found that uncertainty constantly prompted them to refocus on what truly mattered to them. They reflected on their values, with particular emphasis on relationships and on the kind of person they aspired to be. This led them to approach life in a more deliberate and constructive manner.

“Think about your value. Think about what’s important. Think about why people value you. And multiply that out. Be kind to people, even people that you wouldn’t have been, you wouldn’t necessarily have thought about before.” (P9)

Some felt that prostate cancer disrupted their self-concept and identity, prompting a re-evaluation of values, purpose, and past relationships. While not all expressed regret, some reassessed their pre-diagnosis lifestyles, especially in terms of relational engagement. These reflections revealed an evolving sense of self, shaped by emotional awareness and a search for meaning.

“I think one of the aspects about prostate cancer that’s so hard is that it takes away something that’s intrinsically, you.” (P12)

“You think, could I have been a better husband? Could I have been a kinder and more active and more loving husband? Both spiritual and physically than I had been.” (P9)

#### Acceptance.

All but one participant described adopting an accepting attitude toward uncertainty, realising that complete certainty or control was not possible and that the impact of uncertainty were partly shaped by how individuals responded to it.

“I think it’s how you deal with that uncertainty that limits the effect it has on you…. How much you let that uncertainty consume you is how you deal with it. Accept that. If you don’t accept it, I think it’s really going to trouble people.” (P10)

Acceptance was framed as an active willingness to experience reality as it is, rather than as passive resignation. By consciously choosing to let go of what they could not control, such as past regrets or an uncertain future, participants embraced their current experience.

“I’m happy with where I am and my body. My body in many ways doesn’t work in the same way as it used to, but people use this phrase, ‘this is the new reality’.” (P4)

Participants described a range of things they grew to accept, such as the prostate cancer diagnosis, uncertainty about whether they had made the right treatment decision, and the possibility of earlier death.

“I’m not content with the cancer, but I’m more at ease with everything that is around me. I’m more at ease with the fact that I’m going to die early.” (P3)

Being present in the moment was interpreted as a form of acceptance. Shifting toward mindful, present awareness helped participants acknowledge their situations without feeling overwhelmed, promoted emotional regulation, and reduced internal resistance. Over time, this also fostered a sense of progress and positivity.

“Let’s get through today. Let’s get through next week…. If I can do that, that’s good. And then move on to the next week …. As you begin to feel more positive and physically better, I think the uncertainty kind of disappears a bit.” (P4)

#### Agency and control.

Participants described a strong need to regain agency and control. Many distinguished between what was controllable and what was not, and then proactively constructed their future by focusing on things they could exercise control over.

“I was keen to do everything I could do to make the recovery a success…. I’m not in control of cancer coming back, but in my mind, if I look after my health and well-being, then I’m reducing that risk.” (P10)

Some participants began to view themselves as active agents living with cancer rather than passive patients. This shift in self-perception, in conjunction with the intentional actions they chose to take, engendered a greater sense of ownership and control over their cancer-related experience.

“You have to take some kind of ownership for your own health…. it’s very easy just to become a patient where you go from appointment to appointment, you take the pills that they tell you, you do what you’re told. But somehow you gotta try and get back control. And there are so many things that you can’t control, but I started to focus on what I could control.” (P12)

Most participants adopted healthier lifestyles to regain control, such as adjusting to a nutritious diet and increasing physical exercise. This was believed to both enhance their quality of life and reduce the risks of cancer recurrence.

“So I must optimize, if you like, the open-endedness, make it more in my favour by optimizing my life, my diet, my exercise, my relationships, all those things.” (P1)

The need for agency and control also influenced how participants approached support, signposting the significance of person-tailored support. One participant chose to opt out of peer support groups after several attempts, feeling that these groups reinforced a patient identity and a sense of weakness.

“In the end I said, well, why am I going to a patient’s group? So I’m a patient, but do I want that feeling being reinforced all the time? No. …I don’t want to be a patient. Why must I reinforce my sickness as it was by attending a group…. And I decided not to. In the end, it seems to reinforce the negative….” (P1)

Some described proactively learning to live with prostate cancer and, perhaps more importantly, preparing for death, both psychologically and practically. For example, they made plans to ensure family’s quality of life after their passing. These proactive approaches reflected a strong sense of agency and empowerment.

“We’re working on planning for the future. So we’ve done the technical stuff. We’ve got the words written. We’ve done all the stuff that we need to do that will prepare us…. part of that is about making sure that everything is ready when I’m gone.” (P3)

#### Maintain a positive outlook.

All but one participant viewed it as important to maintain an optimistic outlook while addressing uncertainty and other cancer-related challenges.

“I’m just thankful I’m in that right mindset.… The positive mindset is a big part of getting through it.” (P10)

Participants focused on the more positive aspects of situations while acknowledging the negatives. For example, staying alive with undesirable health outcomes (e.g., side effects) was viewed as preferable to the alternative (i.e., death), because it meant there was still hope for improvements.

“Obviously now that I’m post-surgery and I’m still dealing with the erectile dysfunction. It’s on my mind, but I tend to think I don’t have cancer in my body, so I can’t be too disappointed. I just have to be patient and deal with that…. I think in a positive way too. I’m almost like, this is a second chance. I’ve not got the cancer in my body.” (P10)

Engaging in enjoyable activities provided another source of positivity. Participants were grateful to be fit enough to resume some previously enjoyed activities. While some had to reduce or give up certain activities due to physical limitations, they either modified how they approached those activities or discovered new, less physically demanding activities they found enjoyable.

“I have to make a resolution to overcome certain inhibitions in the things I used to do. Not define them by fear. Not say I cannot crack that, but move, try and move into a more uninhibited way of working in the things I’m interested in.” (P1)

Some participants maintained positive outlook by making downward comparisons, comparing themselves to peers facing more serious difficulties or to those less fortunate in other aspects of life.

“Many men don’t have 13 years. Once they find out, they find out too late and they don’t even have 13 weeks, so I’m happy.” (P9)

“You think about the things, you know, other people. Think about people in Gaza, people in Lebanon, people in Israel. You know, there’s disasters in the world. You know, this is small, small things that I had to deal with.” (P7)

Many participants expressed deep appreciation for the love and support they received. Through gratitude, they found strength, perspective, and a sense of control, which helped them stay positive despite uncertainty.

“I’m a great believer in gratitude. Be grateful for what you have in life, whether it’s a roof over your head or a warm bed to go into. Just be grateful, because we’re very lucky.” (P7)

For some, knowing that they would not face challenges alone if their condition worsened provided profound reassurance and a reason to remain positive.

“Now that I don’t know what’s going to happen in the future, but I know that when it does…. we’ve got our own little support network now in place that is helpful to us…. I can cope with most of those things because of the things I’ve got around me. For that I’m truly grateful.” (P3)

Participants also found gratitude in everyday moments. By viewing each day as a gift, they found new layers of hope and reasons not to fear uncertainty. For one participant, gratitude for a life well lived gave him the strength to face death and the unknown without fear.

“I’ve had a good life. I’ve had a happy life. Ups and downs, of course, like everybody. If I was to go next year, I’d be happy. So, I’m not frightened in that respect.” (P9)

#### Support seeking.

All participants noted that openly expressing their concerns, fears, and uncertainty was key to accessing the support they needed. Although seeking support was hard at early stages, they came to recognise their own vulnerability and limitations in facing cancer, and eventually built strong support networks that helped address their informational, emotional and practical needs.

“I found it very difficult to start with. It felt very self-conscious, but I was able to talk about how I felt and was able to ask for help, to be vulnerable, and begin to build a team around me, people who I really trust and I can really be open with.” (P12)

For partnered participants, spouses or partners were often the primary source of support. This included not only practical assistance, such as attending medical appointments or assisting with daily care, but also emotional support and reassurance. Participants stressed the importance of their partners understanding the implications of cancer treatment and its side effects, which often affected them both.

“I’m lucky to be married with a very supportive wife. That’s, you know, definitely been helpful. And she came to all the appointments with me…. It changes things certainly in terms of sex, whether it’s permanent or not remains to be seen. I think possibly not, but there are certainly changes immediately afterwards, but, you know, my wife made it very clear that wasn’t an issue.” (P2)

Most participants found peer support groups, especially those facilitated by specialist nurses or psychologists, to be highly beneficial. Hearing others’ experiences helped them realise that they were not alone in their struggles. These groups also provided a safe, supportive environment where they felt heard, encouraging greater openness and a stronger willingness to seek help.

“That was very interesting. Quite powerful. Sit in a room with people who are going through the same things as you are. You begin to realise you’re not the only person in the world who faces this. And it’s a very calm and safe environment and staffed by trained nurses, so they know what they’re talking about.” (P12)

Participants specifically referred to stories of friends, peers, and public figures to inform their treatment decisions, an area of cancer journey that many found notably uncertain. Learning about the experiences of others who had faced similar challenges provided reassurance and helped validate their choices, reducing decisional uncertainty.

“And listening to his journey, because he was just kind of weeks ahead of where I was, kind of reassured me he had the same surgery, and I guess that was another confirmation that I was making the right decision.” (P10)

Some sought support from healthcare professionals for interpreting medical information, obtaining second opinions, or receiving reassurance. These supports were most effective when tailored to individual needs. For instance, one participant, whose autism further complicated his cancer journey, appreciated that his healthcare providers recognised this and provided additional support that eased treatment-related uncertainty.

“…I was taken through the process of going in a little bit earlier, being shown the facility and the machines, the anxiety had been taken away. The NHS had managed that for me, which was phenomenal…. And they also had made sure that I had them [note: glasses] on when I came back out the sedation. So you know, you weren’t getting sensory deprivation on top of the effect of the general anaesthetic. So I think a lot of the uncertainty around it had been reduced.” (P5)

Two participants sought psychological counselling to address psycho-sexual concerns and emotional distress. Both described the services as immensely helpful.

“…there’s a real need for psychosexual counselling.… I’m still using it…. made a heck of a difference to daily life honestly.” (P5)

### Overarching Theme 4: Posttraumatic growth

Although not prompted, most participants spontaneously described noticing positive changes as a result of navigating uncertainty, aligning with the concept of posttraumatic growth [41].

#### Positive shifts in life perspectives.

Nearly all participants reported having gained new life perspectives. They engaged more in activities that aligned with their reassessed values and life priorities, which in turn deepened their sense of meaning and purpose. These activities included spending quality time with loved ones, volunteering for cancer charities, returning to work, and using their lived experiences of prostate cancer in positive ways.

“Being able to offer guidance and reassurance gives an element of purpose to my experience. This isn’t just about sharing practical advice but also underlining that there can be life - sometimes a richer one - after diagnosis. It’s not an easy road, but this has helped significantly in lifting my own spirits and giving a feeling that I’m part of something meaningful once again.” (P5)

Some felt that coping with cancer and uncertainty led to a deeper self-understanding and a newfound sense of purpose.

“The big positive takeaway for me is self-understanding of those circumstances that over the years have shaped me. They have affected relationships, work, hobbies or whatever. I was always a little aimless…. it helps me to focus it into channels…. I’ve become a lot more focused.” (P8)

Some participants began to see each day as full of possibilities, learning to value the opportunities uncertainty brought rather than fearing that bad things might happen.

“…. look on every day as an opportunity for new experience, to welcome rather than fear each day…. live with each day as a new day and a miracle.” (P1)

One participant described making radical changes to his vocational path due to uncertainty surrounding disease trajectory and life expectancy. These unknowns prompted him to dedicate time and effort to pursuits he deeply enjoyed yet had not dared to pursue before cancer.

“… why would I want to spend what might be the last few years of my life working for a corporation…. Pre-cancer, would I have given my job up to become a DJ? No, no, I wouldn’t have done that. But now I’ve got a different perspective.” (P12)

Some participants became increasingly philosophical, realising that cancer was only one part of life and that life itself was a terminal condition. As a result, they refused to allow cancer to dominate their life and focused instead on what truly mattered.

“We’ve all got a terminal illness. It’s called life. We’re all going to die. It’s just a question of timing…. Decide what’s important, decide what matters, and there’s so much.” (P9)

They acknowledged that uncertainty was an inherent part of life, viewing prostate cancer as merely adding another layer of uncertainty. In light of this, they focused on living life fully.

“We live in uncertain times. Not just the climate emergency, but also strained international relations with threats of war, economic uncertainty and the aftermath of Covid. A diagnosis of stage 4 cancer adds a very personal dimension to this…. Live life as much to the full as I can.” (P1)

#### Enhanced personal strength.

Meaningful personal growth was also reported as a positive change following reflections on the implications of cancer and uncertainty. Some described themselves as more considerate, caring, and emotionally attuned.

“I’m a lot more considerate... I think I’m more caring…. I was probably more selfish in the past…. I just feel a little bit softer in my general mind.” (P11)

Despite previous emotional restraint, participants found themselves becoming increasingly comfortable sharing their inner world, especially when they believed such disclosure might help others.

“I am much more open to discussion about my personal life, whereas before, I was a tightly closed book.” (P8)

After managing prostate cancer-related uncertainty, some reported an improved ability to cope with potential stressors beyond those directly related to the cancer trajectory.

“That was handling the uncertainty for me…. It’s given me new tools to be able to cope with things like pressure.” (P5)

#### Enriching personal relationships.

Positive changes in interpersonal relationships were also identified. Having dealt with difficulties together and experienced personal growth during the process, participants reported having even better, more intimate relationships with their partners.

“…my relationship with my wife became even more closer than it was before because of that experience.” (P4)

Participants also reported strengthened connections with family members and friends, often as a result of changes in their life priorities and personalities.

“I have definitely become more open with loved ones, and surprisingly, this has created stronger connections and greater understanding from family and friends.” (P5)

Following the cancer diagnosis, some participants took on new roles outside their family life. Some of these roles were closely related to prostate cancer, such as volunteering for cancer charities, serving as lay reviewers for cancer research, or contributing to peer support networks. Others were more aligned with their personal skills and expertise. Committing to diverse social roles enriched participants’ interpersonal lives and strengthened their sense of purpose and values.

“I drive the minibus on a Friday. Just between the hotel and the hospital…. And for me, that’s a kind of therapy or catharsis anyway, and it feels like I’m putting something back…. that’s important as well for me to feel that I’m kind of contributing.” (P4)

## Discussion

The present study contributes to the literature by providing novel insights into how men undergoing or having undergone active treatment experienced and navigated uncertainty across the prostate cancer trajectory. Participants described uncertainty related to the disease trajectory, relationships, and the future. Their initial responses to uncertainty were often marked by distress and avoidance. A range of strategies was used to manage uncertainty, including meaning-making, acceptance, efforts to gain agency and control, maintaining a positive outlook, and support-seeking. Overall, our findings show that uncertainty is not merely a cognitive or informational issue but a deeply emotional and relational experience that, when managed adaptively, can become a catalyst for personal transformation.

Uncertainty is a complex and multifaceted experience. It encompassed specific concerns about the disease trajectory, relational uncertainty in interpersonal relationships, and broader existential uncertainty related to the life course and end of life. These dimensions align closely with Mishel’s [2] theory of illness uncertainty. While lack of information was less prominent, complexity, unpredictability, and ambiguity were evident in participants’ accounts. Most participants reported having received adequate medical information to understand the basics of prostate cancer; nevertheless, some sought second opinions or validation of treatment decisions. This revealed lingering uncertainty and an ongoing need to process and integrate information. These findings mirror those in other clinical populations [9] and suggest that the difficulty in addressing uncertainty may lie less in information acquisition and more in its interpretation and integration within one’s personal context.

All participants experienced considerable uncertainty, regardless of cancer stage, treatment modality, or whether primary treatment was complete. Previous research on prostate cancer-related uncertainty has primarily focused on pre-treatment stages or on individuals who had not undergone active treatment [19,23,29–33], which may imply that uncertainty diminishes once treatment ends. Our findings challenge this assumption, showing that uncertainty persists even after active treatment. This is perhaps unsurprising, given the complex interplay of prognostic ambiguity, individual personality differences, and varying coping mechanisms [42–44]. This enduring uncertainty highlights the need for supportive care approaches that address uncertainty as a sustained concern.

Relational uncertainty was identified as a novel and underexplored aspect of uncertainty. It refers to uncertainty surrounding social relationships and emerged as a central feature in our sample. This finding expands the existing conceptualisation of uncertainty in cancer care to include its relational dimensions. While uncertainty is often framed in terms of prognosis, treatment, or informational clarity in health contexts [2,30,45], relational uncertainty may be equally significant. It can interfere with how participants engage with loved ones, negotiate support, and make sense of their changing roles within interpersonal dynamics.

Although our findings reflect only men’s perspectives, relational uncertainty may help explain couples’ shared struggles in adapting to prostate cancer documented in previous research, including mutual avoidance, reduced intimacy, and lower relationship satisfaction [46–49]. These findings suggest that difficulties arising from relational uncertainty may not be adequately addressed by individually focused psychological care. It is therefore important to acknowledge relational uncertainty as an integral part of the prostate cancer experience and to support patients and their partners in navigating relational shifts collaboratively. While our study focused on men with a diagnosis of prostate cancer, the emotional and relational challenges described here may be relevant to other cancer populations where uncertainty is a prominent feature, such as individuals with metastatic disease of unknown primary [7]. Given the limited research into relational uncertainty, future studies should explore how it develops over time, the specific challenges it creates, and how individuals and their social networks manage it effectively.

Uncertainty poses significant challenges for psychological adjustment and requires effective coping. In our study, participants described how uncertainty often triggered psychological distress and avoidant behaviours, such as delayed disclosure of diagnosis or postponed treatment, supporting prior research [4,21,50]. However, as participants began to engage in more adaptive coping, uncertainty was no longer always experienced as negative. One key strategy in this shift was meaning-making, through which men reflected on their cancer experience and sought to integrate it into their broader life narrative. As part of this process, some participants began to reframe uncertainty as a space for hope, self-reflection, and re-evaluation of priorities. This aligns with Mishel’s theory [2], which posits that uncertainty can be appraised both as a threat and as an opportunity. Rather than seeking definitive answers, participants used personal interpretations to derive meaning and coherence. In this sense, uncertainty became a context for psychological resilience, renewed purpose, and personal growth.

Previous research suggests that meaning-making can facilitate psychological adjustment to serious illnesses [51–53], especially when individuals are able to construct coherent and meaningful narratives rather than remain stuck in unproductive ruminations [51]. Cross-sectional and longitudinal studies have similarly found a positive association between successful meaning-making and psychological adjustment among bereaved individuals, people with multiple sclerosis, and their caregivers [54–56], though similar research remains limited in cancer contexts. Our findings contribute to the literature by suggesting that fostering cognitive reappraisal and reframing may not only reduce distress but also promote a sense of coherence and resilience, as reflected in both other strategies men adopted and positive changes they identified.

Acceptance also emerged as an adaptive response, especially given the persistent and often irreducible uncertainty of prostate cancer. Over time, participants reframed uncertainty as an inherent feature of both cancer and life, focusing on how to modify their relationship with it. This acceptance allowed for positivity and progress, consistent with psycho-oncology research linking acceptance-based coping with greater emotional wellbeing [57,58]. Importantly, acceptance and agency were not experienced as mutually exclusive. Many participants pursued agency by focusing on controllable aspects of life, such as making informed decisions or managing their health. Previous quantitative research has highlighted the positive impact of a healthy sense of agency on emotional functioning and quality of life among men with prostate cancer [59,60]. Our results complement this by providing qualitative insight into the pivotal role of agency when facing a highly uncontrollable event like cancer. Specifically, agency and control may be reclaimed through small, deliberate actions that help individuals feel grounded and capable, ultimately supporting greater psychological adjustment.

Maintaining a positive outlook was another approach to managing uncertainty. This involved focusing on positivity and hope and making constructive interpretations of the cancer experience. Importantly, this was not a form of blind optimism in which negative aspects of cancer experience were dismissed. Rather, it reflected men’s deliberate choice to focus more on positive aspects while acknowledging the challenges of their situations. This finding resonates with hope theory, which frames hope as a cognitive process involving goal-directed thinking and action, as well as a sense of agency [61], and psychological flexibility model, which emphasises the adaptive ability to be open to both positive and negative experiences while choosing to act based on personal values [62–64]. Additionally, this strategy is consistent with research highlighting the psychological benefits of hope in cancer populations [65,66].

Participants also commonly reported seeking support from various sources to meet their instrumental, informational, and emotional needs. At first glance, this appears to contrast with previous studies reporting low rates of help-seeking among male clinical populations [67–69], often attributed to adherence to traditional masculine norms such as self-reliance and stoicism [70–74]. One possible explanation is that our recruitment strategy may have attracted individuals who were more open to seeking support. Another is that men with prostate cancer may differ from other male populations in important ways. For example, they may be older, more likely to be in long-term relationships, and possess greater emotional maturity or life experience. These factors may allow for more flexible interpretations of masculine roles, making support-seeking more acceptable, or even adaptive, in the context of illness [72,75,76].

More broadly, men’s narratives of uncertainty in the context of prostate cancer revealed how their experiences were often filtered through masculine ideals and identity. Although the term ‘masculinity’ was not explicitly used, some participants’ initial reactions to uncertainty and subsequent coping were clearly shaped by dominant masculine norms. According to hegemonic masculinity frameworks, these norms emphasise strength, sexual potency, autonomy, and control [77]. For example, some participants initially engaged in avoidant behaviours in response to uncertainty, often driven by a desire not to burden others or appear vulnerable. This reflects adherence to masculine norms of self-reliance and independence and mirrors prior research showing that conforming to such norms can constrain men’s help-seeking and emotional disclosure in health contexts [78,79].

These behaviours may also reflect an underlying need to maintain a positive sense of masculine image or self-worth. From the perspective of Gender Role Strain Theory, failing to live up to internalised masculine ideals can result in identity conflict and psychological strain [80,81]. In our study, participants appeared to manage this strain not by rejecting hegemonic masculine norms outright but by re-evaluating these norms and aligning them with other personally meaningful values. For instance, as interpersonal relationships became more central, they became more emotionally open, more willing to seek help, and more engaged in nurturing interpersonal bonds. These shifts indicate a reworking of masculine norms around self-reliance and stoicism, helping preserve masculine identity.

The patterns observed in the current study suggest that uncertainty is not simply a medical experience, but a gendered one. This provides further support for prior research highlighting the centrality of masculinity in men’s experiences of prostate cancer [72,82]. Masculine identity, while often implicit, appears to shape how men experience and manage prostate cancer-related uncertainty. Engaging with masculinity-related concerns more directly in clinical and research contexts may therefore be key to developing gender-sensitive support that resonates with men’s lived experience.

Although inherently distressing, cancer-related uncertainty may also create space for psychological growth. Several participants spontaneously described positive changes they attributed to their cancer experience, such as an increased appreciation for life or stronger relationships. These accounts align with established models of posttraumatic growth, which propose that growth may arise through the cognitive and emotional processing of traumatic experiences, particularly as individuals work to integrate such experiences into their broader life narrative [41,83–85]. Similar findings have been reported in other populations, including individuals with breast cancer and those receiving palliative care [86,87]. Thornton et al. [88] likewise found that couples coping with prostate cancer experienced growth in terms of personal strength and relational closeness.

Interestingly, although religion and spirituality have been found to support adjustment to cancer [89,90], none of our participants described drawing on religious faith. Similarly, while posttraumatic growth often entails increased spirituality or religious faith [41], no participants described experiencing spiritual growth. This absence may reflect the demographic profile of our sample and broader gender patterns. Prior research has shown that compared to women, men were generally less religious or spiritual and reported fewer spiritual concerns in the context of cancer [91,92]. In addition, most participants in our study had localised prostate cancer, whereas the study identifying spiritual engagement in prostate cancer focused on men with advanced cancer [89]. Existential concerns may be more salient for those facing life-limiting illness, whereas individuals with localised disease may focus more on maintaining daily life and managing side effects. Instead of spiritual reflection, many participants emphasised interpersonal connection, frequently discussing relational uncertainty and the importance of close relationships. Accordingly, the positive changes described were more often located in secular domains, such as increased compassion and strengthened social bonds.

Our findings point to a potentially valuable direction in survivorship care. Specifically, interventions that encourage narrative reflection, values clarification, and acceptance-based practices may help support those who are open to exploring this domain. However, it is important to note that not all men will experience such outcomes, particularly those who are more emotionally distressed or lack external support. Growth should not be assumed or expected, given that the potential for psychological struggle remains significant across the cancer trajectory. Recognising both the potential for growth and the persistence of difficulty is critical for responsive and person-centred care.

## Limitations

These findings need to be interpreted in light of several limitations. First, self-selection bias may have influenced the sample. Individuals comfortable exploring and sharing their inner world may have been more likely to participate, whereas those still struggling with uncertainty or less willing to reflect may have opted out. Second, the study relied on participants’ retrospective accounts, which may have been affected by memory bias or selective recall. Additionally, despite efforts to recruit a diverse sample, the current group was largely homogeneous in terms of ethnicity, sexual orientation, and relationship status. All participants identified as White British and heterosexual, and only one was not in a relationship. Perspectives from single men, sexual minority men, or those from ethnically diverse backgrounds may have differed in meaningful ways and were not captured in this study.

### Clinical implications

Our findings underscore the need for accessible and individually tailored support at multiple stages from diagnosis through to post-treatment. Uncertainty regarding treatment and prognosis signposted the need for decision support, particularly through genuine shared decision-making. This requires medical professionals not only to present and explain treatment options but also to support patients in weighing those options in light of their personal values and preferences [93,94].

Meanwhile, the novel dimension of relational uncertainty highlights the importance of addressing relational dynamics as part of holistic care. Consistent with previous studies [18,48,95], addressing psycho-sexual concerns and renegotiating relationship dynamics were found to be crucial for dyadic adjustment. Healthcare professionals need to be comfortable discussing these concerns with patients and encouraging open conversations within couples. Some couples may particularly benefit from couple-based interventions, such as psycho-sexual counselling or dyadic coping workshops.

To maximise accessibility and improve outcomes, these interventions could be offered at key transitional points (e.g., post-diagnosis consultations or post-treatment reviews) and delivered via multiple formats, including digital platforms, in-person sessions, or mixed approaches. Digital delivery may be especially valuable for older couples facing physical limitations, time constraints, geographical barriers, or limited access to in-person services. Evidence from older populations suggests that digital interventions are particularly effective when tailored to individual needs [96]. Therefore, it may be worthwhile to explicitly adapt the focus of such interventions to reflect each couple’s most pressing concerns (e.g., emotional support versus practical planning). Related to this is the importance of using appropriate assessments to identify relational strain or areas of unmet need within couples.

Finally, the range of coping strategies described by participants may serve as valuable tools in clinical settings. Sharing these strategies with healthcare professionals may enhance their ability to proactively support men’s psychological adjustment and normalise such approaches as part of routine care.

### Suggestions for future research

This study offers important and novel qualitative insights into the experience of uncertainty among men with prostate cancer. Future research should aim to recruit more diverse samples, particularly including men from sexual minority groups, ethnically diverse backgrounds, and a broader range of socioeconomic contexts, to better understand the potentially varied ways in which uncertainty is experienced and navigated. Longitudinal designs following individuals from diagnosis through survivorship would also help clarify how uncertainty evolves over time and how it relates to coping and psychological outcomes. Relational uncertainty, as a novel and largely overlooked dimension, warrants particular attention in this regard.

Future research could also usefully examine how posttraumatic growth develops over time, how it interacts with coping strategies (e.g., whether it results from coping or occurs concurrently), and how it influences psychological outcomes. Such work would enhance understanding of its clinical relevance and inform supportive care practices.

Although existing reviews suggest that Acceptance and Commitment Therapy and Cognitive Behavioural Therapy can benefit cancer populations [97–103], there is limited evidence specific to men with prostate cancer or focused on uncertainty-related outcomes. Future research, such as randomised controlled trials, should investigate the effectiveness of these interventions across different stages of the prostate cancer trajectory. It would also be valuable to explore whether, and how, these approaches foster adaptive meaning-making, tolerance of uncertainty, and acceptance.

Finally, future research should also prioritise the development and evaluation of couple-based interventions that meaningfully address relational uncertainty and support dyadic adjustment. To date, such services are limited and their effectiveness remains inconclusive [104]. A recent meta-analysis found no significant pooled effects of interventions on relationship functioning among couples coping with prostate cancer [105], although individual interventions outperformed conjoint ones. More research is needed to explore the optimal timing, delivery formats, and mechanisms of change to ensure interventions are accessible and meaningful for diverse couples. Given the variability in how couples experience distress, interpret the cancer experience, and engage with psychotherapy [46,47,106], there is a clear need for interventions that can meaningfully address relational uncertainty and foster mutual understanding.

## Supporting information

S1 File Interview protocol. (DOCX)

S2 File Summary table of themes with illustrative quotes. (PDF)

## References

[1] Carleton RN. Into the unknown: A review and synthesis of contemporary models involving uncertainty. J Anxiety Disord. 2016;39:30–43. doi: 10.1016/j.janxdis.2016.02.007 26945765

[2] Mishel MH. Uncertainty in illness. Image J Nurs Sch. 1988;20(4):225–32. doi: 10.1111/j.1547-5069.1988.tb00082.x 3203947

[3] Etkind SN, Barclay S, Spathis A, Hopkins SA, Bowers B, Koffman J. Uncertainty in serious illness: a national interdisciplinary consensus exercise to identify clinical research priorities. PLoS One. 2024;19(2):e0289522. doi: 10.1371/journal.pone.0289522 38422036 PMC10903860

[4] Skojec TA, Davidson TM, Kelechi TJ. The relationship between uncertainty in illness and psychological adjustment to chronic illness. J Health Psychol. 2025;30(4):622–37. doi: 10.1177/13591053241249861 38761058

[5] Verduzco-Aguirre HC, Babu D, Mohile SG, Bautista J, Xu H, Culakova E, et al. Associations of uncertainty with psychological health and quality of life in older adults with advanced cancer. J Pain Symptom Manage. 2021;61(2):369-376.e1. doi: 10.1016/j.jpainsymman.2020.08.012 32822750 PMC7854861

[6] Ghodraty Jabloo V, Alibhai SMH, Fitch M, Tourangeau AE, Ayala AP, Puts MTE. Antecedents and outcomes of uncertainty in older adults with cancer: a scoping review of the literature. Oncol Nurs Forum. 2017;44(4):E152–67. doi: 10.1188/17.ONF.E152-E167 28632247

[7] Argentiero A, Solimando AG, Brunetti O, Calabrese A, Pantano F, Iuliani M, et al. Skeletal metastases of unknown primary: biological landscape and clinical overview. Cancers (Basel). 2019;11(9):1270. doi: 10.3390/cancers11091270 31470608 PMC6770264

[8] Marusyk A, Almendro V, Polyak K. Intra-tumour heterogeneity: a looking glass for cancer? Nat Rev Cancer. 2012;12(5):323–34. doi: 10.1038/nrc3261 22513401

[9] Etkind SN, Bristowe K, Bailey K, Selman LE, Murtagh FE. How does uncertainty shape patient experience in advanced illness? A secondary analysis of qualitative data. Palliat Med. 2017;31(2):171–80. doi: 10.1177/0269216316647610 27129679 PMC5302072

[10] Simonovic N, Taber JM, Scherr CL, Dean M, Hua J, Howell JL, et al. Uncertainty in healthcare and health decision making: five methodological and conceptual research recommendations from an interdisciplinary team. J Behav Med. 2023;46(4):541–55. doi: 10.1007/s10865-022-00384-5 36574173

[11] Brotzman LE, Crookes DM, Austin JD, Neugut AI, Shelton RC. Patient perspectives on treatment decision-making under clinical uncertainty: chemotherapy treatment decisions among stage II colon cancer patients. Transl Behav Med. 2021;11(10):1905–14. doi: 10.1093/tbm/ibab040 34042154 PMC8541697

[12] Lee Y-H, Chou X-Y, Lai Y-H, Liang Y-H, Hung C-T, Hsaio C-C, et al. Decisional conflict and its determinants among patients with cancer undergoing immunotherapy combined with chemotherapy or targeted therapy: a cross-sectional study. Sci Rep. 2023;13(1):12715. doi: 10.1038/s41598-023-39280-6 37543690 PMC10404258

[13] Simard S, Thewes B, Humphris G, Dixon M, Hayden C, Mireskandari S, et al. Fear of cancer recurrence in adult cancer survivors: a systematic review of quantitative studies. J Cancer Surviv. 2013;7(3):300–22. doi: 10.1007/s11764-013-0272-z 23475398

[14] Koch L, Jansen L, Brenner H, Arndt V. Fear of recurrence and disease progression in long-term (≥ 5 years) cancer survivors--a systematic review of quantitative studies. Psychooncology. 2013;22(1):1–11. doi: 10.1002/pon.3022 22232030

[15] Wu S, Guo X, Tang H, Li Y, Dong W, Lu G, et al. The relationship between illness uncertainty and social support among cancer patients: a meta-analysis. Cancer Nurs. 2025;48(5):416–23. doi: 10.1097/NCC.0000000000001328 38447045 PMC12379796

[16] Cancer Research UK. Prostate cancer statistics. Https://WwwCancerresearchukOrg/Health-Professional/Cancer-Statistics/Statistics-by-Cancer-Type/Prostate-Cancer n.d. https://www.cancerresearchuk.org/health-professional/cancer-statistics/statistics-by-cancer-type/prostate-cancer (accessed May 19, 2025).

[17] Rebello RJ, Oing C, Knudsen KE, Loeb S, Johnson DC, Reiter RE, et al. Prostate cancer. Nat Rev Dis Primers. 2021;7(1):9. doi: 10.1038/s41572-020-00243-0 33542230

[18] Varner S, Lloyd G, Ranby KW, Callan S, Robertson C, Lipkus IM. Illness uncertainty, partner support, and quality of life: a dyadic longitudinal investigation of couples facing prostate cancer. Psychooncology. 2019;28(11):2188–94. doi: 10.1002/pon.5205 31418505

[19] Parker PA, Davis JW, Latini DM, Baum G, Wang X, Ward JF, et al. Relationship between illness uncertainty, anxiety, fear of progression and quality of life in men with favourable-risk prostate cancer undergoing active surveillance. BJU Int. 2016;117(3):469–77. doi: 10.1111/bju.13099 25714186 PMC4547910

[20] Pan S, Wang L, Zheng L, Luo J, Mao J, Qiao W, et al. Effects of stigma, anxiety and depression, and uncertainty in illness on quality of life in patients with prostate cancer: a cross-sectional analysis. BMC Psychol. 2023;11(1):129. doi: 10.1186/s40359-023-01159-6 37098648 PMC10131473

[21] Guan T, Santacroce SJ, Chen D, Song L. Illness uncertainty, coping, and quality of life among patients with prostate cancer. Psycho-Oncology. 2020;29(6):1019–25. doi: 10.1002/pon.5372 32128938 PMC7440775

[22] Eisenberg SA, Kurita K, Taylor-Ford M, Agus DB, Gross ME, Meyerowitz BE. Intolerance of uncertainty, cognitive complaints, and cancer-related distress in prostate cancer survivors. Psychooncology. 2015;24(2):228–35. doi: 10.1002/pon.3590 24891013

[23] Wade J, Donovan J, Lane A, Davis M, Walsh E, Neal D, et al. Strategies adopted by men to deal with uncertainty and anxiety when following an active surveillance/monitoring protocol for localised prostate cancer and implications for care: a longitudinal qualitative study embedded within the ProtecT trial. BMJ Open. 2020;10(9):e036024. doi: 10.1136/bmjopen-2019-036024 32907896 PMC7482454

[24] Smith DP, Supramaniam R, King MT, Ward J, Berry M, Armstrong BK. Age, health, and education determine supportive care needs of men younger than 70 years with prostate cancer. J Clin Oncol. 2007;25(18):2560–6. doi: 10.1200/JCO.2006.09.8046 17577034

[25] Dunn J, Ralph N, Green A, Frydenberg M, Chambers SK. Contemporary consumer perspectives on prostate cancer survivorship: Fifty voices. Psychooncology. 2020;29(3):557–63. doi: 10.1002/pon.5306 31944447

[26] Moore THM, King AJL, Evans M, Sharp D, Persad R, Huntley AL. Supportive care for men with prostate cancer: why are the trials not working? A systematic review and recommendations for future trials. Cancer Med. 2015;4(8):1240–51. doi: 10.1002/cam4.446 25828811 PMC4559035

[27] Paterson C, Robertson A, Smith A, Nabi G. Identifying the unmet supportive care needs of men living with and beyond prostate cancer: a systematic review. Eur J Oncol Nurs. 2015;19(4):405–18. doi: 10.1016/j.ejon.2014.12.007 25613370

[28] Schildmeijer K, Frykholm O, Kneck Å, Ekstedt M. Not a straight line-patients’ experiences of prostate cancer and their journey through the healthcare system. Cancer Nurs. 2019;42(1):E36–43. doi: 10.1097/NCC.0000000000000559 29334523

[29] Bailey DE, Mishel MH, Belyea M, Stewart JL, Mohler J. Uncertainty intervention for watchful waiting in prostate cancer. Cancer Nurs. 2004;27(5):339–46. doi: 10.1097/00002820-200409000-00001 15525860

[30] Bailey DE Jr, Wallace M, Mishel MH. Watching, waiting and uncertainty in prostate cancer. J Clin Nurs. 2007;16(4):734–41. doi: 10.1111/j.1365-2702.2005.01545.x 17402955

[31] Kazer MW, Bailey DE Jr, Sanda M, Colberg J, Kelly WK. An Internet intervention for management of uncertainty during active surveillance for prostate cancer. Oncol Nurs Forum. 2011;38(5):561–8. doi: 10.1188/11.ONF.561-568 21875843

[32] Mishel MH, Germino BB, Lin L, Pruthi RS, Wallen EM, Crandell J, et al. Managing uncertainty about treatment decision making in early stage prostate cancer: a randomized clinical trial. Patient Educ Couns. 2009;77(3):349–59. doi: 10.1016/j.pec.2009.09.009 19819096

[33] Wallace M. Uncertainty and quality of life of older men who undergo watchful waiting for prostate cancer. Oncol Nurs Forum. 2003;30(2):303–9. doi: 10.1188/03.onf.303-309 12692664

[34] Green R. Experiences and management of uncertainty following treatment for prostate cancer. Health Risk Soc. 2024;26(5–6):240–59. doi: 10.1080/13698575.2024.2345631

[35] Braun V, Clarke V. Thematic analysis: a practical guide. SAGE Publications Ltd. 2021.

[36] Pietkiewicz I, Smith JA. A practical guide to using Interpretative Phenomenological Analysis in qualitative research psychology. CPPJ. 2014;20(1). doi: 10.14691/cppj.20.1.7

[37] Sim J, Saunders B, Waterfield J, Kingstone T. Can sample size in qualitative research be determined a priori? Int J Soc Res Methodol. 2018;21(5):619–34. doi: 10.1080/13645579.2018.1454643

[38] Vasileiou K, Barnett J, Thorpe S, Young T. Characterising and justifying sample size sufficiency in interview-based studies: systematic analysis of qualitative health research over a 15-year period. BMC Med Res Methodol. 2018;18(1):148. doi: 10.1186/s12874-018-0594-7 30463515 PMC6249736

[39] Malterud K, Siersma VD, Guassora AD. Sample size in qualitative interview studies: guided by information power. Qual Health Res. 2016;26(13):1753–60. doi: 10.1177/1049732315617444 26613970

[40] Tong A, Sainsbury P, Craig J. Consolidated criteria for reporting qualitative research (COREQ): a 32-item checklist for interviews and focus groups. Int J Qual Health Care. 2007;19(6):349–57. doi: 10.1093/intqhc/mzm042 17872937

[41] Tedeschi RG, Calhoun LG. TARGET ARTICLE: “Posttraumatic growth: conceptual foundations and empirical evidence”. Psychol Inq. 2004;15(1):1–18. doi: 10.1207/s15327965pli1501_01

[42] Bergengren O, Pekala KR, Matsoukas K, Fainberg J, Mungovan SF, Bratt O, et al. 2022 update on prostate cancer epidemiology and risk factors—a systematic review. Eur Urol. 2023;84(2):191–206. doi: 10.1016/j.eururo.2023.04.021 37202314 PMC10851915

[43] Sanda MG, Dunn RL, Michalski J, Sandler HM, Northouse L, Hembroff L, et al. Quality of life and satisfaction with outcome among prostate-cancer survivors. N Engl J Med. 2008;358(12):1250–61. doi: 10.1056/NEJMoa074311 18354103

[44] Vince RA Jr, Jiang R, Bank M, Quarles J, Patel M, Sun Y, et al. Evaluation of social determinants of health and prostate cancer outcomes among Black and White patients: a systematic review and meta-analysis. JAMA Netw Open. 2023;6(1):e2250416. doi: 10.1001/jamanetworkopen.2022.50416 36630135 PMC9857531

[45] Mishel MH. The measurement of uncertainty in illness. Nurs Res. 1981;30(5):258–63. doi: 10.1097/00006199-198109000-00002 6912987

[46] Manne S, Badr H, Zaider T, Nelson C, Kissane D. Cancer-related communication, relationship intimacy, and psychological distress among couples coping with localized prostate cancer. J Cancer Surviv. 2009;4(1):74–85. doi: 10.1007/s11764-009-0109-y 19967408 PMC2828868

[47] Song L, Northouse LL, Zhang L, Braun TM, Cimprich B, Ronis DL, et al. Study of dyadic communication in couples managing prostate cancer: a longitudinal perspective. Psychooncology. 2012;21(1):72–81. doi: 10.1002/pon.1861 20967920 PMC3875561

[48] Collaço N, Rivas C, Matheson L, Nayoan J, Wagland R, Alexis O, et al. Prostate cancer and the impact on couples: a qualitative metasynthesis. Support Care Cancer. 2018;26(6):1703–13. doi: 10.1007/s00520-018-4134-0 29511952

[49] Badr H, Taylor CLC. Sexual dysfunction and spousal communication in couples coping with prostate cancer. Psychooncology. 2009;18(7):735–46. doi: 10.1002/pon.1449 19061199 PMC4476400

[50] Ahadzadeh AS, Sharif SP. Uncertainty and quality of life in women with breast cancer: moderating role of coping styles. Cancer Nurs. 2018;41(6):484–90. doi: 10.1097/NCC.0000000000000552 29489477

[51] Park CL. Making sense of the meaning literature: an integrative review of meaning making and its effects on adjustment to stressful life events. Psychol Bull. 2010;136(2):257–301. doi: 10.1037/a0018301 20192563

[52] Park CL, Edmondson D, Fenster JR, Blank TO. Meaning making and psychological adjustment following cancer: the mediating roles of growth, life meaning, and restored just-world beliefs. J Consult Clin Psychol. 2008;76(5):863–75. doi: 10.1037/a0013348 18837603

[53] Gillies J, Neimeyer RA. Loss, grief, and the search for significance: toward a model of meaning reconstruction in bereavement. J Constr Psychol. 2006;19(1):31–65. doi: 10.1080/10720530500311182

[54] Keesee NJ, Currier JM, Neimeyer RA. Predictors of grief following the death of one’s child: the contribution of finding meaning. J Clin Psychol. 2008;64(10):1145–63. doi: 10.1002/jclp.20502 18698614

[55] Pakenham KI. Making sense of illness or disability: the nature of sense making in multiple sclerosis (MS). J Health Psychol. 2008;13(1):93–105. doi: 10.1177/1359105307084315 18086721

[56] Pakenham KI. Making sense of multiple sclerosis. Rehabil Psychol. 2007;52(4):380–9. doi: 10.1037/0090-5550.52.4.380

[57] Secinti E, Tometich DB, Johns SA, Mosher CE. The relationship between acceptance of cancer and distress: a meta-analytic review. Clin Psychol Rev. 2019;71:27–38. doi: 10.1016/j.cpr.2019.05.001 31078056 PMC7010402

[58] Czerw A, Religioni U, Deptała A. Assessment of pain, acceptance of illness, adjustment to life with cancer and coping strategies in breast cancer patients. Breast Cancer. 2016;23(4):654–61. doi: 10.1007/s12282-015-0620-0 26031432 PMC4911383

[59] Helgeson VS, Lepore SJ. Quality of life following prostate cancer: the role of agency and unmitigated agency1. J Applied Social Pyschol. 2004;34(12):2559–85. doi: 10.1111/j.1559-1816.2004.tb01992.x

[60] Helgeson VS, Lepore SJ. Men’s adjustment to prostate cancer: the role of agency and unmitigated agency. Sex Roles. 1997;37(3–4):251–67. doi: 10.1023/a:1025651912128

[61] Snyder CR. Hope theory: rainbows in the mind. Psychological Inquiry. 2002;13(4):249–75. doi: 10.1207/s15327965pli1304_01

[62] Hayes SC, Luoma JB, Bond FW, Masuda A, Lillis J. Acceptance and commitment therapy: model, processes and outcomes. Behav Res Ther. 2006;44(1):1–25. doi: 10.1016/j.brat.2005.06.006 16300724

[63] Hayes SC, Villatte M, Levin M, Hildebrandt M. Open, aware, and active: contextual approaches as an emerging trend in the behavioral and cognitive therapies. Annu Rev Clin Psychol. 2011;7(1):141–68. doi: 10.1146/annurev-clinpsy-032210-104449 21219193

[64] Kashdan TB, Disabato DJ, Goodman FR, Doorley JD, McKnight PE. Understanding psychological flexibility: a multimethod exploration of pursuing valued goals despite the presence of distress. Psychol Assess. 2020;32(9):829–50. doi: 10.1037/pas0000834 32614192

[65] Berendes D, Keefe FJ, Somers TJ, Kothadia SM, Porter LS, Cheavens JS. Hope in the context of lung cancer: relationships of hope to symptoms and psychological distress. J Pain Symptom Manag. 2010;40(2):174–82. doi: 10.1016/j.jpainsymman.2010.01.014 PMC2921459 20579840

[66] Oztunc G, Yesil P, Paydas S, Erdogan S. Social support and hopelessness in patients with breast cancer. Asian Pac J Cancer Prev. 2013;14(1):571–8. doi: 10.7314/apjcp.2013.14.1.571 23534797

[67] Ettridge KA, Bowden JA, Chambers SK, Smith DP, Murphy M, Evans SM, et al. “Prostate cancer is far more hidden…”: Perceptions of stigma, social isolation and help-seeking among men with prostate cancer. Eur J Cancer Care. 2017;27(2):e12790. doi: 10.1111/ecc.12790 29112317

[68] Fish JA, Prichard I, Ettridge K, Grunfeld EA, Wilson C. Psychosocial factors that influence men’s help-seeking for cancer symptoms: a systematic synthesis of mixed methods research. Psychooncology. 2015;24(10):1222–32. doi: 10.1002/pon.3912 26202128

[69] Rice SM, Aucote HM, Parker AG, Alvarez-Jimenez M, Filia KM, Amminger GP. Men’s perceived barriers to help seeking for depression: Longitudinal findings relative to symptom onset and duration. J Health Psychol. 2017;22(5):529–36. doi: 10.1177/1359105315605655 26391789

[70] Wall D, Kristjanson L. Men, culture and hegemonic masculinity: understanding the experience of prostate cancer. Nurs Inq. 2005;12(2):87–97. doi: 10.1111/j.1440-1800.2005.00258.x 15892724

[71] Oliffe JL. Connecting masculinities to men’s illness vulnerabilities and resilience. Qual Health Res. 2023;33(14):1322–32. doi: 10.1177/10497323231198967 37902085 PMC10666520

[72] Goodwin BC, Ralph N, Ireland MJ, Hyde MK, Oliffe JL, Dunn J, et al. The role of masculinities in psychological and emotional help seeking by men with prostate cancer. Psychooncology. 2020;29(2):356–63. doi: 10.1002/pon.5264 31659799

[73] Wong YJ, Ho M-HR, Wang S-Y, Miller ISK. Meta-analyses of the relationship between conformity to masculine norms and mental health-related outcomes. J Couns Psychol. 2017;64(1):80–93. doi: 10.1037/cou0000176 27869454

[74] Addis ME, Mansfield AK, Syzdek MR. Is “masculinity” a problem?: Framing the effects of gendered social learning in men. Psychol Men Masc. 2010;11(2):77–90. doi: 10.1037/a0018602

[75] Harden J. Developmental life stage and couples’ experiences with prostate cancer: a review of the literature. Cancer Nurs. 2005;28(2):85–98. doi: 10.1097/00002820-200503000-00002 15815178

[76] Bowie J, Brunckhorst O, Stewart R, Dasgupta P, Ahmed K. Body image, self-esteem, and sense of masculinity in patients with prostate cancer: a qualitative meta-synthesis. J Cancer Surviv. 2022;16(1):95–110. doi: 10.1007/s11764-021-01007-9 33963973 PMC8881246

[77] Connell RW, Messerschmidt JW. Hegemonic masculinity: rethinking the concept. Gender Soc. 2005;19(6):829–59. doi: 10.1177/0891243205278639

[78] Corboy D, Meier J, McLaren S. Self-reliance and stoicism as predictors of distress following radical prostatectomy in the context of place of residence. Psychol Men Masculinities. 2019;20(4):637–46. doi: 10.1037/men0000197

[79] Seidler ZE, Dawes AJ, Rice SM, Oliffe JL, Dhillon HM. The role of masculinity in men’s help-seeking for depression: a systematic review. Clin Psychol Rev. 2016;49:106–18. doi: 10.1016/j.cpr.2016.09.002 27664823

[80] Levant RF, Richmond K. The gender role strain paradigm and masculinity ideologies. APA handbook of men and masculinities. American Psychological Association; 2016. p. 23–49. doi: 10.1037/14594-002

[81] Pleck JH. The gender role strain paradigm: an update. In: Levant RF, Pollack WS, editors. A new psychology of men. New York: Basic Books/Hachette Book Group; 1995. p. 11–32.

[82] Chambers SK, Chung E, Wittert G, Hyde MK. Erectile dysfunction, masculinity, and psychosocial outcomes: a review of the experiences of men after prostate cancer treatment. Transl Androl Urol. 2017;6(1):60–8. doi: 10.21037/tau.2016.08.12 28217451 PMC5313306

[83] Tedeschi RG, Calhoun LG. The posttraumatic growth inventory: Measuring the positive legacy of trauma. J Traumatic Stress. 1996;9(3):455–71. doi: 10.1002/jts.2490090305 8827649

[84] Hefferon K, Grealy M, Mutrie N. Post-traumatic growth and life threatening physical illness: a systematic review of the qualitative literature. Br J Health Psychol. 2009;14(Pt 2):343–78. doi: 10.1348/135910708X332936 18718109

[85] Caspari JM, Raque-Bogdan TL, McRae C, Simoneau TL, Ash-Lee S, Hultgren K. Posttraumatic growth after cancer: the role of perceived threat and cognitive processing. J Psychosoc Oncol. 2017;35(5):561–77. doi: 10.1080/07347332.2017.1320347 28414581

[86] Austin PD, Siddall PJ, Lovell MR. Posttraumatic growth in palliative care settings: a scoping review of prevalence, characteristics and interventions. Palliat Med. 2024;38(2):200–12. doi: 10.1177/02692163231222773 38229018

[87] Huang S, Huang M, Long F, Wang F. Post-traumatic growth experience of breast cancer patients: a qualitative systematic review and meta-synthesis. PLoS One. 2025;20(1):e0316108. doi: 10.1371/journal.pone.0316108 39847563 PMC11756777

[88] Thornton AA, Perez MA. Posttraumatic growth in prostate cancer survivors and their partners. Psychooncology. 2006;15(4):285–96. doi: 10.1002/pon.953 16035136

[89] Lepherd L. Spirituality in men with advanced prostate cancer: “it’s a holistic thing . . . it’s a package”. J Holist Nurs. 2014;32(2):89–101; quiz 102–3. doi: 10.1177/0898010113504492 24080341

[90] Paul A, Kalir T. The role of spirituality/religiosity in the cancer journey. MRAJ. 2025;13(3). doi: 10.18103/mra.v13i3.6318

[91] Rassoulian A, Gaiger A, Loeffler-Stastka H. Gender differences in psychosocial, religious, and spiritual aspects in coping: a cross-sectional study with cancer patients. Womens Health Rep (New Rochelle). 2021;2(1):464–72. doi: 10.1089/whr.2021.0012 34841392 PMC8617579

[92] Hvidt NC, Mikkelsen TB, Zwisler AD, Tofte JB, Assing Hvidt E. Spiritual, religious, and existential concerns of cancer survivors in a secular country with focus on age, gender, and emotional challenges. Support Care Cancer. 2019;27(12):4713–21. doi: 10.1007/s00520-019-04775-4 30963295

[93] Stiggelbout AM, Van der Weijden T, De Wit MPT, Frosch D, Légaré F, Montori VM, et al. Shared decision making: really putting patients at the centre of healthcare. BMJ. 2012;344:e256. doi: 10.1136/bmj.e256 22286508

[94] Elwyn G, Frosch D, Thomson R, Joseph-Williams N, Lloyd A, Kinnersley P, et al. Shared decision making: a model for clinical practice. J Gen Intern Med. 2012;27(10):1361–7. doi: 10.1007/s11606-012-2077-6 22618581 PMC3445676

[95] Chambers SK, Schover L, Nielsen L, Halford K, Clutton S, Gardiner RA, et al. Couple distress after localised prostate cancer. Support Care Cancer. 2013;21(11):2967–76. doi: 10.1007/s00520-013-1868-6 23756617

[96] Riadi I, Kervin L, Dhillon S, Teo K, Churchill R, Card KG, et al. Digital interventions for depression and anxiety in older adults: a systematic review of randomised controlled trials. Lancet Healthy Longev. 2022;3(8):e558–71. doi: 10.1016/S2666-7568(22)00121-0 36102765

[97] Fang P, Tan L, Cui J, Yu L. Effectiveness of Acceptance and Commitment Therapy for people with advanced cancer: a systematic review and meta-analysis of randomized controlled trials. J Adv Nurs. 2023;79(2):519–38. doi: 10.1111/jan.15543 36534441

[98] Jiang X, Sun J, Song R, Wang Y, Li J, Shi R. Acceptance and commitment therapy reduces psychological distress in patients with cancer: a systematic review and meta-analysis of randomized controlled trials. Front Psychol. 2024;14:1253266. doi: 10.3389/fpsyg.2023.1253266 38250124 PMC10796538

[99] Maunick B, Skvarc D, Olive L, Mikocka-Walus A. Effects of acceptance and commitment therapy on fatigue for patients with cancer and other chronic health conditions: a systematic review and meta-analysis. J Psychosom Res. 2023;171:111366. doi: 10.1016/j.jpsychores.2023.111366 37270911

[100] Li H, Wu J, Ni Q, Zhang J, Wang Y, He G. Systematic review and meta-analysis of effectiveness of acceptance and commitment therapy in patients with breast cancer. Nurs Res. 2021;70(4):E152–60. doi: 10.1097/NNR.0000000000000499 33492055

[101] Xia W, Zheng Y, Guo D, Zhu Y, Tian L. Effects of cognitive behavioral therapy on anxiety and depressive symptoms in advanced cancer patients: a meta-analysis. Gen Hosp Psychiatry. 2024;87:20–32. doi: 10.1016/j.genhosppsych.2024.01.006 38280276

[102] Zhang L, Liu X, Tong F, Zou R, Peng W, Yang H, et al. Cognitive behavioral therapy for anxiety and depression in cancer survivors: a meta-analysis. Sci Rep. 2022;12(1):21466. doi: 10.1038/s41598-022-25068-7 36509786 PMC9744858

[103] Zhao C, Lai L, Zhang L, Cai Z, Ren Z, Shi C, et al. The effects of acceptance and commitment therapy on the psychological and physical outcomes among cancer patients: a meta-analysis with trial sequential analysis. J Psychosom Res. 2021;140:110304. doi: 10.1016/j.jpsychores.2020.110304 33248396

[104] Chambers SK, Pinnock C, Lepore SJ, Hughes S, O’Connell DL. A systematic review of psychosocial interventions for men with prostate cancer and their partners. Patient Educ Couns. 2011;85(2):e75-88. doi: 10.1016/j.pec.2011.01.027 21334159

[105] Ma H, Yang Y, Li Y, Cariola L, Gillanders D. Effectiveness of psychological interventions in improving relationship functioning among couples coping with prostate cancer: a systematic review and meta‐analysis. Psycho-Oncology. 2025;34(1). doi: 10.1002/pon.70080 PMC11728261 39804293

[106] Manne SL, Kashy D, Myers‐Virtue S, Zaider T, Kissane DW, Heckman CJ, et al. Relationship communication and the course of psychological outcomes among couples coping with localised prostate cancer. Eur J Cancer Care. 2021;30(4). doi: 10.1111/ecc.13401 PMC9165020 33586282

