# Supplementary material for: Experience of uncertainty in prostate cancer: A qualitative study
Source: PLoS One. 2025 Oct 13;20(10):e0334180. doi: 10.1371/journal.pone.0334180 (PMC12517514; doi:10.1371/journal.pone.0334180)
Supplement: S1 File — (DOCX) [file pone.0334180.s001.docx]

Introduction

Thank you for agreeing to take part in this research. We are interested in men’s experience of uncertainty while living with prostate cancer.

We expect the interview to last between 45 and 60 minutes, but we can stop to take a break at any point, and you can finish the interview at any time without giving an explanation.

[If participant has consented to be recorded, switch on the recorder].

May I double-check that you understand your research rights as described in the consent form and that you’re willing to take part in this interview? May I double-check that you are happy for this interview to be recorded?

[Continue recording if participant has confirmed their consent.]

**Interview questions**

1. Please can you tell me a bit about your general experience of prostate cancer?

2. Some people report feeling uncertainty at various points since diagnosis, is this something you have ever experienced?

3. Can you tell me what kinds of uncertainty you came across during the cancer journey? Maybe you would like to think about specific events that created uncertainty during the timeframe of your journey.

4. What effects did those uncertainties have upon you?

5. Among the uncertainties you just talked about, are some forms of uncertainties more distressing than others? If so, which ones do you think are the most distressing or upsetting?

6. What happens internally when there’s uncertainty emerging? What kinds of thoughts or feelings emerge?

7. How do you tend to respond when there are uncertainties as well as the relating thoughts and feelings?

8. What do you think are the reasons why you tend to respond in the ways that you just described?

9. What consequences do you notice of when you respond in the ways that you just described?

10. What do you find particularly helpful in dealing effectively with distress relating to uncertainty?

11. How important do you think the ability to tolerate or accept uncertainty is for people with prostate cancer?

12. Are there any resources that you hope to have for you to better cope with uncertainties during the journey? (If no, what resources would you recommend to others for others recently diagnosed with prostate cancer to accept and cope with uncertainties?)

Closing question

Is there anything else you would like to discuss that has not already been covered?
