# Supplementary material for: Experience of uncertainty in prostate cancer: A qualitative study
Source: PLoS One. 2025 Oct 13;20(10):e0334180. doi: 10.1371/journal.pone.0334180 (PMC12517514; doi:10.1371/journal.pone.0334180)
Supplement: S2 File — (PDF) [file pone.0334180.s002.pdf]

Theme	Subtheme	Examples of codes	Illustrative quotes
Theme 1. Aspects of uncertainty	1.1 Uncertainty disease trajectory	1.1.1 Uncertainty regarding diagnosis	Limited or lack of knowledge regarding prostate:<br>“The uncertainty at the beginning was I couldn't have told you what my prostate did.” (P9)<br><br>Little knowledge regarding test specifics:<br>“I couldn't have told you what prostate cancer meant. I couldn't have told you what a PSA score was. I didn't even know that PSA scores indicated the likelihood of the extent of cancer.” (P9)<br><br>Uncertainty regarding the meaning of a diagnosis:<br>“I know what stage three means. What does the B mean?” (P8)<br><br>Uncertainty regarding the implications of cancer or whether cancer is curable:<br>“I thought I was gonna die imminently because I just got no insight into what the cancer was.” (P3)
		1.1.2 Uncertainty regarding treatment options	Unsure how a treatment is defined as successful:<br>“The unknown is what are you coming out as after it? Because you can never tell how well that surgery is going to go. Yeah.” (P10)<br><br>Uncertain regarding whether one’s treatment was successful:<br>“Rightly or wrongly, I made a decision to go for robotic assisted removal.” (P5)<br><br>“What you can never be sure is what the outcome's going to be. How successful it's going to be.” (P10)<br><br>The sort of advice I got at the time was that there's a 50-50 chance that that will fix the cancer and kill it.” (P3)<br><br>Lack of knowledge of specific treatment modality:<br>“I've been sort of told about hormone therapy. I've no idea what that was, but it didn't really

Theme	Subtheme	Examples of codes	Illustrative quotes
			sound too bad. But I had really no idea whatsoever as to what that was and what that involved." (P12)<br><br>"The one thing I did really struggle with was the fact it was going to be a general anaesthetic…. am I going to die under this? You know, what's it? So all of that worry was there." (P5)
		1.1.3 Uncertainty regarding adverse effects	Uncertainty regarding the risks of side effects:<br>"I felt very uncomfortable because of a lack of data. And then I kept asking, what are the risks? …. I think there was a lack of clear presentation from oncology about the risks and about what I was facing." (P12)<br><br>Uncertainty regarding what had caused side effects:<br>"I was given wrong advice about catheters, whether that caused the problem, I don't know." (P2)<br><br>Uncertainty regarding how life would be affected by side effects:<br>"I've been chemically castrated. I was told that from day one. So one of the big questions is how does that affect me? I had a normal sexual life. I have a female partner who's still physically active. Very. So how do we deal with that?" (P1)<br><br>"I guess my biggest concern was probably the incontinence, thinking how can I go offshore and take a month's worth of pads and things offshore and dispose of them." (P10)<br><br>Uncertainty regarding how long side effects would last:<br>"It changes things certainly in terms of sex, whether it's permanent or not remains to be seen." (P2)<br><br>"But when you get round to the sort of, you know, the things you're used to having as a man each morning when you, that's not there, so you know, they say to you that might not be there for three months, four months. It's completely false. You know the answer is it might not be there for two to five years and only under certain circumstances, and you may not

Theme	Subtheme	Examples of codes	Illustrative quotes
			ever." (P5)
		1.1.4 Uncertainty regarding cancer prognosis	Uncertainty regarding cancer progression:<br>"So what's gonna happen? Where's the cancer going to spread?" (P3)<br><br>"Part of that is dealing with the specific uncertainty of the progression of the disease." (P1)<br><br>Uncertainty regarding life expectancy:<br>"I don't know how far it goes. And I hear of stories of people surviving 10 years and more on the treatment I'm having. So you get to an unknown." (P1)<br><br>Uncertainty regarding cancer recurrence:<br>"I guess there is still that lingering feeling of doubt, could this (cancer) come back?" (P10)<br><br>"If the surgery didn't get all the cancer out, you can go on then and have radiotherapy or brachytherapy or possibly even chemotherapy. So there was that kind of fall-back position that if the surgery hadn't been 100% successful, I could then go on to another set of treatments. Whereas if you go for the brachytherapy, you can't then do surgery, you know so that was another part of the decision making." (P7)

Theme	Subtheme	Examples of codes	Illustrative quotes
	1.2 Relational uncertainty	1.2.1 Uncertainty regarding intimate relationship stability	Uncertainty regarding whether partners fully understand the implications of living with cancer and provide support as needed:<br>“So in terms of intimate relationships, um, you need to, it's an issue for the partner to understand. And my partner does and doesn't, but does in part. Yeah. So how do we resolve and find new ways, you know, of relating physically. And that's still an ongoing struggle.” (P1)<br><br>Fears of burdening others and/or restricted self-disclosure and communication due to this:<br>“Because I think they will panic a bit. I think when they hear the word cancer, it will send all the alarm bells off and everything, and there's nothing really achieved by them.” (P6)<br><br>“I think it could be a very upsetting conversation for both and if truth be known, I'm probably as much of a coward as many men, and if I had the courage, I would go straight in and say it, but I'm not sure I do have at the moment. I will, but I'm not ready to do that yet.” (P9)<br><br>“Maybe we sort of skirted around that subject. I should have said, but you know, how do you feel about this? Shouldn't I? Oh dear. Life lesson.” (P11)<br><br>“When I got the diagnosis with prostate cancer, all I felt was I anger with myself because I put everybody I love in a position where they had to worry.” (P8)
		1.2.3 Uncertainty regarding chance of forming intimate relationship	“So I was single when I caught the diagnosis. So you think to yourself, well, you're single forever, like you know who's gonna want to have a relationship with me now.” (P12)
		1.2.4 Uncertain interpersonal relationships in broader social contexts (e.g., workplace)	Uncertainty regarding whether one would be seen or treated differently by others:<br>“You don't know how other people are going to react.” (P9)<br><br>“Can people see this in me, you know? So do people know I'm different?” (P5)
	1.3 Uncertainty about what the	1.3.1 Unpredictability of long-term health and quality of life	A form of existential uncertainty:<br>“I don’t know how far it goes. And I hear of stories of people surviving 10 years and more

Theme	Subtheme	Examples of codes	Illustrative quotes
	future holds		on the treatment I'm having. So you get to an unknown.” (P1)<br><br>“I've had good treatment. I wonder how long I will last.” (P10)
		1.3.2 Tension between hope and reality	Side effects lasted longer than hoped:<br>“I had been doing pelvic floor exercises and so on before the operation for a month or two and did them religiously after, but the incontinence didn't go…. So in a way, what’s the killer was the hope there that things would be better. And then of course, at each stage when it wasn't better. So, there’s gap between your expectations and, you know, the reality. Yeah.” (P2)<br><br>“there are days when you really just plummet mentally, as a consequence of the surgery, because you get up and you think. Nothing's happening yet, and you're still waiting for that…. I mean, I've often said, you know, would you do it again? No, I might select watchful waiting because I've had 10 years more, you know, normal life…. Because you think I've reached that landmark that they've set out as being a possible, you know, and nothing's happening at all.” (P5)<br><br>Unexpected cancer recurrence:<br>“I was expecting him to say because all the tests up to that point had been normal and the GP had said no, there's nothing. There's nothing wrong. Your PSA number is 4. Your examination of your atrophy is fine. Nice and smooth. No problems. But because of your family history and I thought this was just a routine. It's a word, you know, it's just something you go through, and the guy says you're fine. Off you go, you know, but he didn't,” (P4)<br><br>“But then suddenly, nine months later, my PSA score suddenly shot up.” (P9)<br><br>Unexpected psychological pathway (more challenging than believed):<br>“…when you lose something as important and as light as central to your life as sexual function and the psychology that goes with that, then at first you think, well, I'm going to get through this. I'm going to carry on. I'm still a man. I'm still going to deep voice. I'm still me. But it starts to eat away. Funnily enough, it starts to eat away at your mind, and you start to

Theme	Subtheme	Examples of codes	Illustrative quotes
			think, could have done better. So at the end of Schindler's List, you know, I could have saved more. I could have done better. And that has had an increasingly profound effect on me.” (P9)
		1.3.3 Difficulties in planning into future	Difficulties planning into the future (in general sense):<br>“I find it difficult to plan into the future. I set medium term goals, reach them, and have to think again…. Very difficult to think beyond short term horizons.” (P1)<br><br>“Because through the surgery and the radiotherapy and all the different treatments you tend to see very short time distances ahead, a day, a week maybe, but nothing beyond.” (P4)<br><br>Difficulties relating to make financial plans:<br>“if I've got 10 years to live, we can do this, this, this and this and this. You know, we can go on holidays, we can take the kids out there, we can give some money or whatever else it is. But if I've got 20 years left to live, plus I might have some care home at the end of my life, what does that mean financially? And it's the great unknown, isn't it? I know you asked about a health question, but to me the financial and the health thing are so inextricably linked. When you get to a certain age, it gets very weird. You know, the age does very strange things to the way that you think about things.” (P11)
Theme 2. Reactions to uncertainty	2.1 Negative emotions	2.1.1 Shock or disbelief	“I was diagnosed about three years ago. It was a shock, profound shock.” (P4)<br><br>“When I heard those words, you have medium aggressive cancer. And I wasn't expecting it.” (P4)<br><br>“I don't want to underestimate kind of a shock it was to be told you had prostate cancer. I guess in, you know, July, August 2016, you know, I must have found it a challenge, you know, looking back. Oh my goodness.” (P7)
		2.1.2 Sadness	“And I was just, I remember, just crying in the car, really. Almost like a big release. You know, I can't even think why. If I was scared? Then I called my wife and let her know and then we drove home. And I remember the day that was absolute torrential rain, I thought, well, I gotta stop crying or I won't be able to see where I'm going. So yeah, that part was

Theme	Subtheme	Examples of codes	Illustrative quotes
			almost like a process." (P10)<br><br>"I guess the feeling of loss, and regret, I think that goes into your personal life when you lose sexual function. You think, could I have been a better husband? Could I have been a kinder and more active and more loving husband?" (P9)
		2.1.3 Anxiety	"There'd still be the anxiety of cancer in the background…. I just knew that cancer kills people and so it caused a lot of anxiety, a lot of distress." (P3)<br><br>"Probably in the early tests. I was probably slightly anxious that, you know waiting for the result and hoping it would be fine." (P7)
		2.1.4 Anger	"But the initial diagnosis didn't have me fearful at all. I was just really angry with myself because I'd let myself take it that far." (P8)<br><br>"And then that become anger after a while, when you realise, no, I haven't been told the truth here." (P5)
		2.1.5 Fear or Hopelessness	"The treatments. I was frightened. I was very, very frightened about radiotherapy. And as I went through the process of radiotherapy, and it got worse and worse and the side effects of it got worse and worse and worse. I just didn't see an end point and it was a horrible" (P3)<br><br>"The psychological aspect might be interesting. I don't know if I was afraid to hear what it might be, but I never felt that or whether or not…. I don't know why I ignored it subconsciously, perhaps fear." (P8)<br><br>"…quite often you go to the worst-case scenario." (P4)<br><br>"The uncertainty during the night was I couldn't do anything about that." (P5)
	2.2. Avoidance	2.2.1 delay in seeking medical attention (late diagnosis)	"I knew there was something wrong. I knew there was something wrong three years before diagnosis, but I just said, well, it'll go away. It'll just go away." (P8)
		2.2.2 delayed treatment	"So that process of deciding what to do took about four months. And I think, yeah, felt that

Theme	Subtheme	Examples of codes	Illustrative quotes
			was unnecessarily long. And nobody really benefited from that because that delayed my treatment and I think there was a lack of clear presentation from oncology about the risks and about what I was facing.” (P12)
		2.2.3 Rumination	“Psychologically it changed my life because I was preoccupied with thinking about it every day. I was thinking about it all the time. It was constant thinking and ruminating on something that I didn't want to have.” (P3)<br><br>“They do make you spend time thinking about it. A lot. People probably think you're daydreaming, but you're actually thinking about all the "what if" questions.” (P9)
Theme 3. Managing uncertainty	3.1 Meaning making	3.1.1 Reflections on implications of pre-existing values and past experience	“I work offshore … So yeah. I think that's where the resilience and strength come from. Your mindset of dealing with that.” (P10)<br><br>“I have gone through my life thinking that everything and anything that goes wrong for me, for my family or around me is my fault. And that's the reason that I think I've wanted to seek affirmation and conformation that people think I'm doing all right. And I think that's informed my attitude to cancer.” (P9)<br><br>“Faced with the diagnosis of an incurable cancer with an unknown survival prognosis, I felt an impulse to address some of the unresolved issues in my life.” (P1)<br><br>“You think, could I have been a better husband? Could I have been a kinder and more active and more loving husband? Both spiritual and physically than I had been.” (P9)
		3.1.2 Positive reframing	“Fortunately, I am able to lead a normal life.” (P1)<br><br>“I thought, you know, I've got quite a long time ahead of me, if I get this right. I would quite like down the line to have my prostate working again…to give myself a bit more drive, give myself a bit more energy and everything else. And I think that was partly at the root of it.” (P11)<br><br>“It's not necessarily been at all negativities by any means. There is a positive aspect to this

Theme	Subtheme	Examples of codes	Illustrative quotes
			process that I've been through….. I allow myself to have these moments of uncertainty and moments of introspection and moments of pessimism, if you like. But that can't determine what your life is going to be like….” (P4)
		3.1.3 Focus on what matters	“Think about your value. Think about what's important. Think about why people value you. And multiply that out. Be kind to people, even people that you wouldn't have been, you wouldn't necessarily have thought about before.” (P9)
		3.1.4 Reappraisal of loss of self	“I think one of the aspects about prostate cancer that's so hard is that it takes away something that's intrinsically, you.” (P12)<br><br>“You think, could I have been a better husband? Could I have been a kinder and more active and more loving husband? Both spiritual and physically than I had been.” (P9)<br><br>“I've never been in a situation where this made me feel, you know, that in any way sort of affected my dignity. But it is what it is.” (P6)
	3.2 Acceptance	3.2.1 Acceptance is important	“Again, I think that was like a really important thing for me that I was trying to accept there were a lot of things I couldn't control…. I think you have to be able to accept the reality.” (P3)<br><br>“In a sense, you don't have a choice. Well, you do have choices either of explained, but you know the reality of it.” (P4)<br><br>“But yeah, so that's the components. It's what it is, yeah. Yeah, right.” (P6)<br><br>“I think you have to be able to accept the reality.” (P12)<br><br>“… how much you let that uncertainty consume you is how you deal with it. Accept that. If you don't accept it, I think it's really going to trouble people.” (P10)
		3.2.2 Experience reality as it is	“…it's also learning because you're not able to do everything you did before because you're, you know, physically weaker and it takes a bit to get your strength back.” (P5)

Theme	Subtheme	Examples of codes	Illustrative quotes
			"I'm happy with the where I am and my body. My body in many ways doesn't work the same way as it used to, but people use this phrase, 'this is the new reality'." (P4)<br><br>"I knew it was going to be a decision that would change my life, but once, you know, being pragmatic, once the decisions made and I knew that's the course we were going to go." (P9)
		3.2.3 Be present in the moment	"let's get through today. Let's get through next week. But not any further than that. If I can do that, that's good. And then move on to the next week and the next week and the next week. As you begin to feel more positive and physically better, I think the uncertainty kind of disappears a bit." (P4)
	3.3 Agency and control	3.3.1 Focus on the controllable	"I was keen to do everything I could do to make the recovery a success." (P10)<br><br>"I'm not in control of cancer coming back, but in my mind, if I look after my health and well-being, then I'm reducing that risk." (P10)<br><br>"you're just trying to focus on the areas of your life that you can control and you can improve." (P12)
		3.3.2 Active agent, rather than passive patient	"…you have to take some kind of ownership for your own health… But I felt that really helped me because it was my own decision, so I wasn't being pushed by someone to do something. I had made my own decision and I think that helped me a lot." (P12)<br><br>"…maybe because things don't happen automatically, you have to construct your future, you know? Um, I'm struggling with that one. Because without those things, I will lose a certain positivity and energy of moving forward." (P1)<br><br>"you have to be clear yourself, don't you? As to why you think a course of action suits you better; if you just made a stat decision and gone, yeah, I think I'll go that way. If you can't defend your position in a proper discussion, then you know that that's awkward." (P11)
		3.3.3 Adopting a healthy lifestyle	"I want to do what I do, which is eat well…. I changed my diet actually." (P6)

Theme	Subtheme	Examples of codes	Illustrative quotes
			"I also had the nutritionist. So that obviously was much more narrow in terms of advice, but I got a tremendous amount from that psychologically and physically." (P12)<br><br>"I need to look after my diet and exercise and health and keep myself fit, not to kind of just ignore. It's made me more sensitive to my body." (P10)
		3.3.4 Informed choice of support sources	"but I haven't really been bothered about joining sort of support groups. I don't know. I mean I think you need to pick people you know are gonna be supportive." (P6)<br><br>"I know some people find those groups very helpful, but it didn't work for me particularly." (P1)
		3.3.5 Prepare for future (e.g.,death)	"I've been on a close group at Maggie's with one of the specialists there and a couple of other ladies, and that's about living with cancer. But basically, it's about preparing for death, I guess. And it's given us insights into life when you have got an incurable disease." (P3)
	3.4 Stay positive and maintain hope	3.4.1 Importance of positive mindset	"And you don't, you no longer see it as quite as threatening. It's inconvenient and it's not very pleasant to go through. But again, it's not the end of the world." (P4)<br><br>"They say you get a cancer diagnosis and if you worry about it, it seems to exacerbate the problem and you get worse and worse and worse." (P8)
		3.4.2 Focus on positives while acknowledging negatives	"Obviously now that I'm post-surgery and I'm still dealing with the erectile dysfunction. It's on my mind, but I tend to think I don't have cancer in my body. I can't be too disappointed. I just have to be patient and deal with that….I think in a positive way too. I'm almost like, this is a second chance. I've not got the cancer in my body. OK?" (P7)
		3.4.3 Engage in enjoyable activities	"I went to YouTube. Found old essential selection mixers by DJs that I listened to in my teens. That was the one place that I got some energy, some positivity, some relief. And I just listened to those over and over again." (P12)<br><br>"the children generally are happy and they like to be with me. I enjoy being with them. They enjoy being with me. So let's deal with that. Let's keep it at that just now and we'll talk about the other stuff later, if I need to." (P4)

Theme	Subtheme	Examples of codes	Illustrative quotes
		3.4.4 Make downward comparisons	“You think about the things, you know, other people. Think about people in Gaza, people in Lebanon, people in Israel. You know, there's disasters in the world. You know, this is small, small things that I had to deal with.” (P7)<br><br>“Many men don’t have 13 years. Once they find out, they find out too late and they don’t even have 13 weeks, so I’m happy.” (P9)
		3.4.5 Gratitude towards things that provide reason for staying positive	“I’m a great believer in gratitude. Be grateful for what you have in life, whether it’s a roof over your head or a warm bed to go into. Just be grateful, because we’re very lucky.” (P7)<br><br>“I’ve had a good life. I’ve had a happy life. Ups and downs, of course, like everybody. If I was to go next year, I’d be happy. So I’m not frightened in that response.” (P9)
		3.4.6 Medical advancements	“So part of living on is always in the hope. There's going to be some cure as opposed to some management of the disease. So you have your eye on that all the time. So how do you keep your eye on the thing, be aware of it.” (P1)
	3.5 Support seeking	3.5.1 Importance of revealing vulnerability	“And if you don't talk about it and you don't face up to the effect it's having on you, it will eat away. And that's what it's been doing, I think.” (P9)<br><br>“I was able to talk about how it felt and able to have enough vulnerability to ask for help and to be vulnerable, and to begin to build a team around me, people who I really trust and I can really be open with.” (P12)
		3.5.2 Spousal support	“My wife has been hugely supportive. You know, we're together all the time.” (P11)<br><br>“I think it's my wife who's been the brave one in supporting me, looking after me, making sure I'm OK, making sure I have a nap in the afternoon, if I'm tired, or whatever, so I’m not alone.” (P4)<br><br>“She then sort of took it back to the basics and said what I'm going to do is document this because she needed something to help her understand and you know, she started writing that and then obviously she was doing the same as you're doing. So it's sitting down. Talk to me about this. Talk to me about that. So, that was great, because what we did was we got all

Theme	Subtheme	Examples of codes	Illustrative quotes
			things in and we did it on the basis of not a list of things, but trying to do it from here's what it is, here's how it manifests. And here's how you can handle it.” (P5)<br><br>“I mean again my wife is supporting me well. Her support on me has just been really good. So she's been like a real star with that.” (P6)<br><br>“my wife was with me every single appointment” (P7)
		3.5.3 Peer support	“I've got a particular friend, [name], who had colon cancer a few years ago and he and I have chatted a couple of times when I've felt I don't know what happens next. I phoned him or I've been to see him and we had a cup of tea and we just chat. Nobody has all the answers, but just talking to somebody solves a lot of the problems yourself while you're speaking.” (P11)<br><br>“I've joined a couple of support groups, the cancer club, which is an online support group, and that's got a prostate cancer sort of division. And on LinkedIn there are loads and loads and loads of guys who've got prostate cancer and we all talk to one another. And that helps quite a lot actually.” (P8)
		3.5.4 Support on treatment decision-making	“That was the other one I did when I was making the decision about my treatment, Kenny Logan, the ex Scotland rugby international. And listening to his journey, because he was just kind of weeks ahead of where I was. Kind of reassured me he had the same surgery and I guess that was another confirmation that I was making the right decision.” (P10)<br><br>“Because once people know that you you've got this condition, they say, oh my friend's got that, my friend would like to talk to you and then people who've had it years ago, say, God, I wish I'd gone for radiotherapy, and then people who've gone for radiotherapy and then it's come back, they’re like, God. I wish I'd had it removed. So there's no right or wrong or anything in this.” (P11)
		3.5.5 Healthcare professionals support	“I have a lot of fantastic supports from the urology department at the hospital, Prostate cancer UK. There's a load of people out there if you're feeling down, you can phone and ask for advice” (P4)

Theme	Subtheme	Examples of codes	Illustrative quotes
			"…I was taken through the process of going in a little bit earlier, being shown the facility and the machines, the anxiety had been taken away. The NHS had managed that for me, which was phenomenal…. And they also had made sure that I had them [note: glasses] on when I came back out the sedation. So you know, you weren't getting sensory deprivation on top of the effect of the general anaesthetic. So I think a lot of the uncertainty around it had been reduced." (P5)
		3.5.6 Psychological professional support	"I'm now no longer PTSD because we went through it all…. So that's been the glorious thing that's come out of the prostate cancer experience really….And that's why there's a real need for psychosexual counselling coming out at the end of these sort of things." (P5)<br><br>"She's very good. I'm personally finding it very helpful in exploring why I am the way that I am and what's important to me and why I crave approval." (P9)
Theme 4. Posttraumatic growth	4.1 Positive shifts in life perspectives	4.1.1 Meaningful activities aligning with reassessed values	"Cancer forced me to reassess many things. Now, I find myself appreciating simple moments: my afternoon latte after a drive to the local farm shop, helping my daughter and son with their projects, being able to do a walk with the dog. These aren't just pleasant distractions; they've become meaningful." (P5)
		4.1.2 Greater self-understanding and new sense of purpose	"I understand myself better now and I understand how much self-criticism and why I've been so hard on myself…. being kinder to myself is not self-indulgent and not selfish. It's the key to mental health. And I think I've lost track of that altogether completely. But I think that cancer has amplified all of that. 'Cause, you're gonna have a limited life, so there's pressure on you now to do something about this. You don't know how long you're gonna be around." (P9)
		4.1.3 New life possibility	"So what do you do with the knowledge you have? Um, how does it change your thinking? And it took me a long time to begin to think a bit like Sam Rushdi and his recent publication, the Knife, you try to live with each day as a new day and a miracle." (P1)<br><br>"Pre-cancer, would I have given my job up to become a DJ? No, no, I wouldn't have done that. But now I've got a different perspective." (P12)
		4.1.4 Increasingly	"We've all got a terminal illness. It's called life. We're all going to die. It's just a question of

Theme	Subtheme	Examples of codes	Illustrative quotes
		philosophical	timing…. Decide what's important, decide what matters, and there's so much.” (P9)<br><br>“We live in uncertain times. Not just the climate emergency, but also strained international relations with threats of war, economic uncertainty and the aftermath of Covid. A diagnosis of stage 4 cancer adds a very personal dimension to this…. Live life as much to the full as I can.” (P1)
	4.2 Enhanced personal strength	4.2.1 Positive personality changes	“I'm a lot more considerate... I think I'm more caring…. I was probably more selfish in the past…. I just feel a little bit softer in my general mind.” (P11)
		4.2.2 More open or self-disclosure	“I am much more open to discussion about my personal life, whereas before, I was a tightly closed book.” (P8)
		4.2.3 Increased coping skills	“That was handling the uncertainty for me…. It's given me new tools to be able to cope with things like pressure.” (P5)
	4.3 Enriching personal relationships	4.3.1 More intimate relationship with partner	“…my relationship with my wife became even more closer than it was before because of that experience” (P4)
		4.3.2 Greater connections with family and friends	“I have definitely become more open with loved ones, and surprisingly, this has created stronger connections and greater understanding from family and friends.” (P5)
		4.3.3 Diverse social roles	“I drive the minibus on a Friday. Just between the hotel and the hospital…. And for me, that's a kind of therapy or catharsis anyway, and it feels like I'm putting something back…. that's important as well for me to feel that I'm kind of contributing.” (P4)
